# Single-Molecule Conductance Behavior of Molecular Bundles

**DOI:** 10.1021/acs.inorgchem.3c01943

**Published:** 2023-12-11

**Authors:** Alejandro Bara-Estaún, Inco J. Planje, Renad Almughathawi, Saman Naghibi, Andrea Vezzoli, David C. Milan, Colin Lambert, Santiago Martin, Pilar Cea, Richard J. Nichols, Simon J. Higgins, Dmitry S. Yufit, Sara Sangtarash, Ross J. Davidson, Andrew Beeby

**Affiliations:** † Department of Chemistry, Durham University, South Rd, Durham DH1 3LE, U.K.; ‡ Department of Chemistry, University of Liverpool, Crown St, Liverpool L69 7ZD, U.K.; § Department of Physics, Faculty of Science, Taibah University, Madinah 42353, Saudi Arabia; ∥ Department of Physics, University of Lancaster, Lancaster LA1 4YB, U.K.; ⊥ Instituto de Nanociencia y Materiales de Aragón (INMA), CSIC-Universidad de Zaragoza, 50009 Zaragoza, Spain; # Departamento de Química Física, Universidad de Zaragoza, 50009 Zaragoza, Spain; ∇ Laboratorio de Microscopias Avanzadas (LMA), Universidad de Zaragoza, 50018 Zaragoza, Spain; ○ School of Engineering, University of Warwick, Coventry CV4 7AL, U.K.

## Abstract

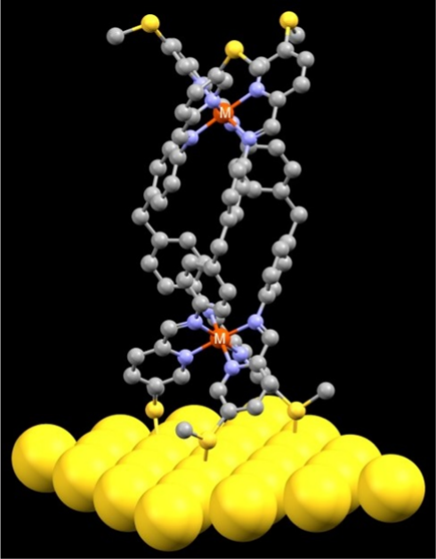

Controlling the orientation of complex molecules in molecular junctions is crucial to their development into functional devices. To date, this has been achieved through the use of multipodal compounds (i.e., containing more than two anchoring groups), resulting in the formation of tri/tetrapodal compounds. While such compounds have greatly improved orientation control, this comes at the cost of lower surface coverage. In this study, we examine an alternative approach for generating multimodal compounds by binding multiple independent molecular wires together through metal coordination to form a molecular bundle. This was achieved by coordinating iron(II) and cobalt(II) to 5,5′-bis(methylthio)-2,2′-bipyridine (**L**^**1**^) and (methylenebis(4,1-phenylene))bis(1-(5-(methylthio)pyridin-2-yl)methanimine) (**L**^**2**^) to give two monometallic complexes, **Fe-1** and **Co-1**, and two bimetallic helicates, **Fe-2** and **Co-2**. Using XPS, all of the complexes were shown to bind to a gold surface in a *fac* fashion through three thiomethyl groups. Using single-molecule conductance and DFT calculations, each of the ligands was shown to conduct as an independent wire with no impact from the rest of the complex. These results suggest that this is a useful approach for controlling the geometry of junction formation without altering the conductance behavior of the individual molecular wires.

## Abstract 2

A combined synthetic, XPS, conductive STM, and computation study that demonstrates how multiple molecular wires can be bundled by metal coordination without significantly impacting the conductance behavior of the individual wire.

## Introduction

Single-molecule conductance determination has become a primary tool in molecular electronics since it provides valuable direct insights into the transport of charge through individual molecules.^1−5^ This has prompted the investigation of increasingly complex molecules and molecular assemblies as a way of achieving new electrical functionality of molecular junctions. However, as molecular junction complexity increases, the defined orientation and anchoring of molecular assemblies can become challenging. Here, the orientation of the molecule with respect to the substrate becomes critical in achieving defined junction morphology that maps onto a consistent and reproducible electrical response.

Current attempts toward improving molecular junction definition involve producing multipodal compounds with more than two contact groups or using contacts with very specific binding geometries. An emerging approach utilizes tri/tetrapodal compounds, where the backbone of the conductor is connected by multiple anchors to the substrate, with each contact being either electronically coupled or insulated from the conductive path.^6−11^ In both cases, improved junction formations have been observed due to increased interaction with the electrode. However, the addition of multiple contact groups results in a large footprint, resulting in a lower surface coverage. Lower surface coverage may not be desirable since high surface coverage can promote better packing and ordering in self-assembled monolayers (SAMs) and this might better protect large-area molecular devices from short-circuiting as a result of the more stable and contiguous molecular monolayers. Therefore, several considerations such as stable and defined surface anchoring, reproducible electrical response, and surface assembly and stability are all important. In achieving these attributes, while developing new electrical functions, it is worthwhile exploring new methods to tether and assemble molecular wires within electrical junctions.

An alternative approach to assembling and tethering molecular junctions is to bundle multiple simple conductors together, thereby promoting a higher conductor density through geometric control. Shen et al. recently demonstrated that it is possible to join two parallel *p*-quaterphenyl molecular wires, which resulted in a slight increase in conductance (attributed to a through-space contribution).^12^ This study seeks to expand on Shen’s work by combining three conductors into a bundle in a modular fashion; metallosupramolecular chemistry provides a convenient route for doing this. If each of the ligands used to build the complex structure is conjugated, then the addition of anchor groups produces molecular wires. One of the simplest of these is a 5,5′-substituted-2,2′-bipyridine. Although no conductance studies have been reported on 5,5′-substituted-2,2′-bipyridine complexes, previous work by Ponce et al. demonstrated that 3,8-substituted 1,10-phenanthroline (structurally similar to 5,5′-substituted-2,2′-bipyridine) showed little difference in conductance behavior when coordinated to a transition metal, which suggests that in this motif, the primary conductance path does not include the metal.^13^ However, in the case of [2,2′-bipyridine]-4,4′-diamine-based complexes, where conductance occurs through the metal–pyridine bond, the metal ion dictates the conductive behavior.^14^ In the work reported in this manuscript, iron(II) and cobalt(II) complexes were synthesized as both metal ions are labile and octahedral, giving isostructural complexes. When conductance occurs through a pyridine–cobalt(II) path, a significantly higher conductance is achieved relative to the analogous iron(II);^15^ this observation provides a suitable evaluation of whether charge flow through the molecule is occurring through the metal center of the bundle.

## Results and Discussion

### Synthesis

**Scheme 1:**
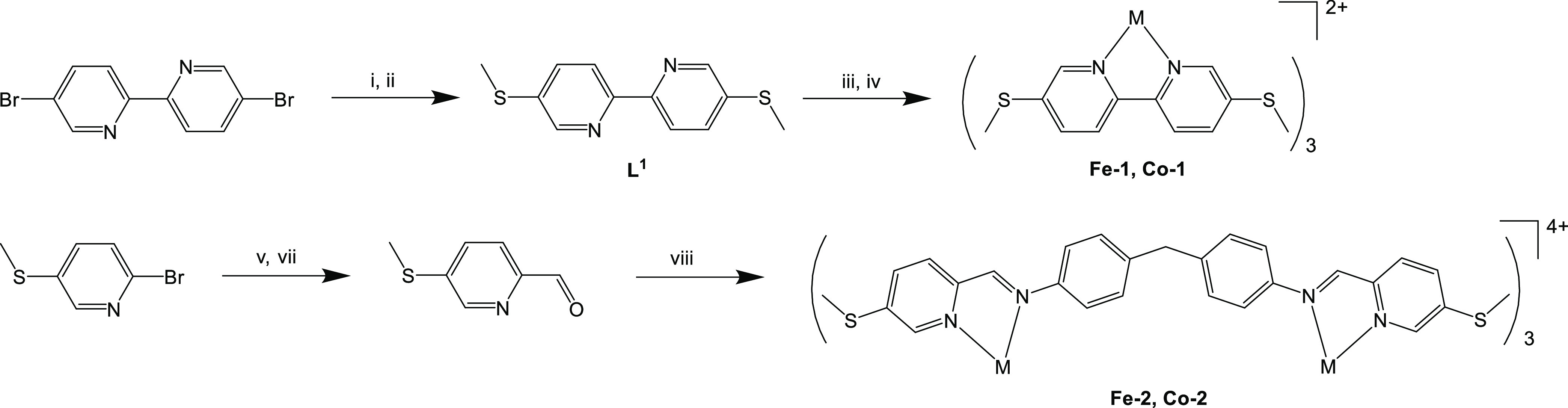
Synthesis of **L**^**1**^ and Complexes **Fe-1, Co-1**, **Fe-2**, **Co-2**; (i) *n*BuLi, (ii) Dimethyldisulfide, (iii) M(BF_4_)_2_·6H_2_O, Where M = Co(II) or Fe(II), (iv) NH_4_PF_6_, (v) *n*BuLi, (vi) DMF, (vii) 4,4′-Methylenedianiline and M(BF_4_)_2_·6H_2_O, Where M = Co(II) or Fe(II)

5,5′-Bis(methylthio)-2,2′-bipyridine (**L**^**1**^) was synthesized by double-lithiating 5,5′-dibromo-2,2′-bipyridine (bpy-Br_2_) by using 2.5 equiv of nBuLi, followed by the addition of dimethyldisulfide (see Scheme 1). The use of 1 equiv of nBuLi resulted in a mixture of bpy-Br_2_ and the product, with no monosubstituted species present, which suggests that upon first lithiation, the molecule is activated to a second. The **L**^**1**^ was reacted with Fe(BF_4_)_2_·6H_2_O or Co(BF_4_)_2_·6H_2_O to give the corresponding iron(II) (**Fe-1**) and cobalt(II) (**Co-1**) complexes, with corresponding yields of 72 and 55%.

As a means of testing the limits of junction stability, analogous complexes were examined with a high aspect ratio; therefore, a bimetallic metallohelicate was employed, based on the work of Hannon. The iron(II) (**Fe-2**) and cobalt(II) (**Co-2**) helicates were produced by first lithiating 2-bromo-5-(methylthio)pyridine, followed by treatment with DMF, to give the corresponding 5-(methylthio)picolinaldehyde. This was reacted with 4,4′-methylenedianiline to form the imine (**L**^**2**^) in situ,^16^ followed by the addition of a metal salt in a single-pot reaction. These reactions proceeded rapidly, with purification achieved by crystallization to give yields of **Fe-2** (81%) and **Co-2** (68%). It is noteworthy that although **L**^**2**^ was formed in the absence of the metal salts, it could not be satisfactorily purified as the free ligand.

### Molecular Structures

**Figure 1:**
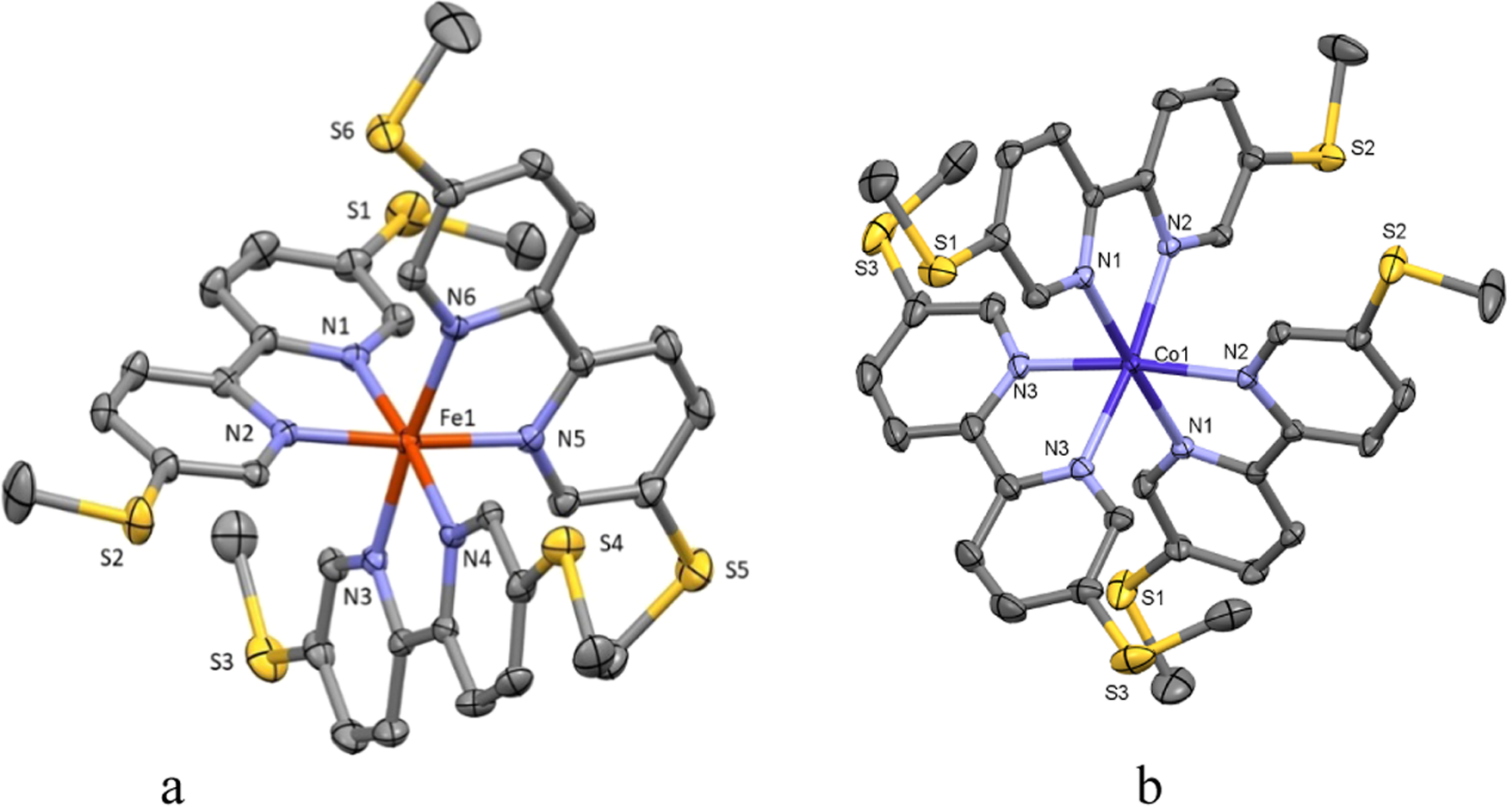
Cation of **Fe-1**(a) and **Co-1** (b) in crystal; Hydrogen atoms, anions, and solvent molecules removed for clarity; thermal ellipsoids displayed at 50% probability.

**Figure 2:**
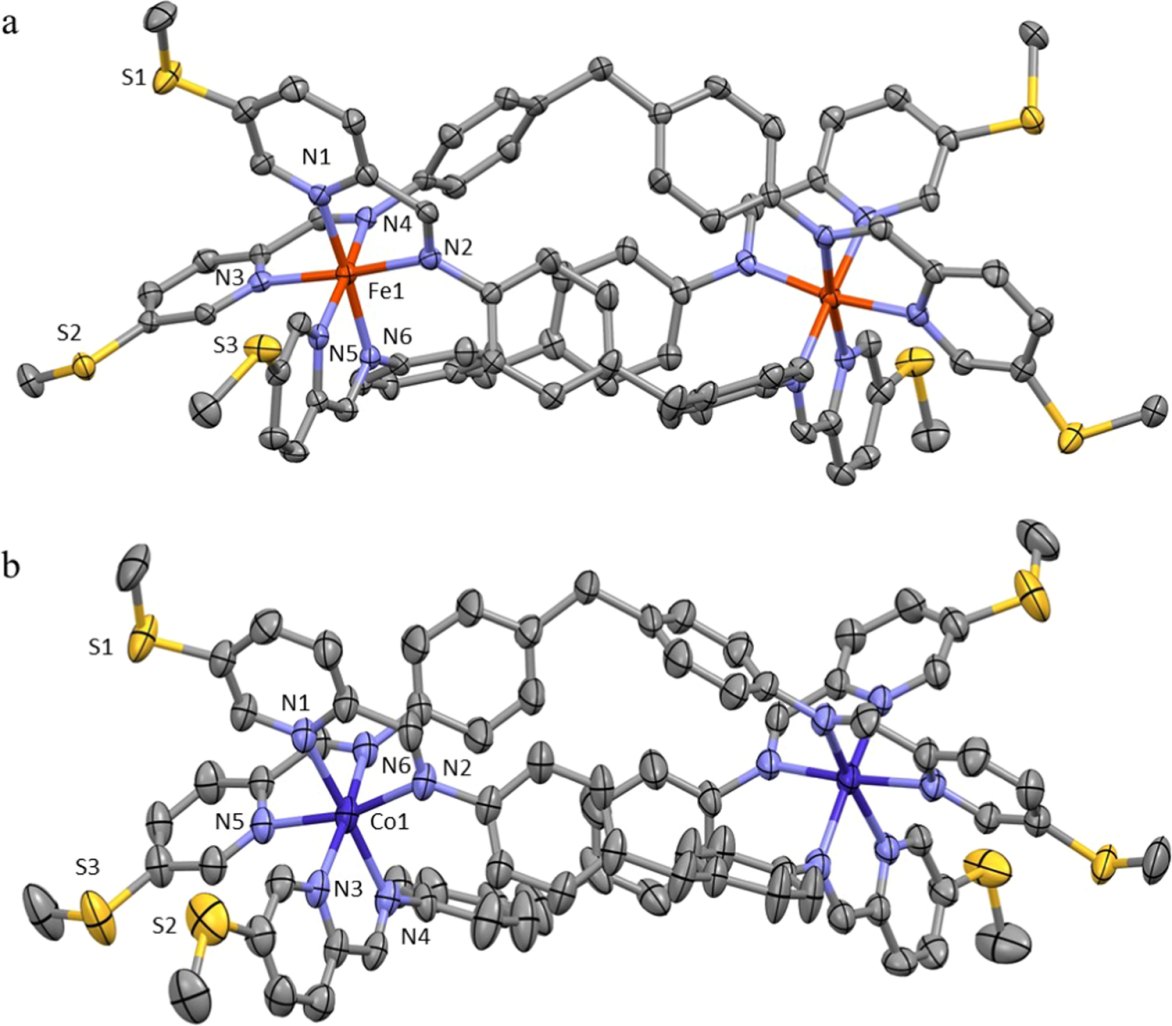
Cation of (a) **Fe-2** and (b) **Co-2** in crystal (H-atoms, anions, and solvent molecules removed for clarity; thermal ellipsoids displayed at 50% probability).

Crystal structures of complexes **Fe-1** and **Co-1** showed typical tribipyridine complexes with a slightly distorted octahedral coordination environment of the metal centers (Figure 1), with **Co-1** showing a marginally higher distortion of octahedral geometry around the metal atom (minimum N–Co–N angle 76.62 vs 81.82° in **Fe-1**, and average M–N bond lengths of 2.130 Å **Co-1** vs 1.916 Å **Fe-1**) than in **Fe-1**. This was attributed to **Co-1** being in the high-spin (HS) state, whereas **Fe-1** was in the low-spin (LS) state.^17^ This difference in geometries resulted in a more compact shape of cation **Fe-1** in comparison with **Co-1**, which results in a closer spatial arrangement of S atoms in **Fe-1**: the range of S···S distances there varied from 6.349 to 6.618 Å, while in the **Co-1** complex, all of these distances were longer than 7.2 Å. Iron(II)-based helicate complexes have been described before,^18^ and the geometry of the cation **Fe-2** corresponded well to previously observed ones. As observed from **Fe-1** and **Co-1**, the average M–N bond lengths were 1.979 Å (**Fe-2**) and 2.137 Å (**Co-2**), which was consistent with the former being LS and the latter being HS. The distance between the S_3_ planes at the opposite sides of the cation was 19.435(2) Å. The S···S distances in **Co-2** (6.349–6.720 Å) were shorter than in **Co-1**, probably due to the higher flexibility of the ligands. That led to a similar columnar packing arrangement to that found in **Fe-2**, with the distance between the opposite S_3_-planes in **Co-2** being 20.065(2) Å (Figure 2).

### XPS Data/Surface Coverage Data

In order to gain insights into the binding interactions between complexes and a gold substrate via the various potential thiol contacting groups, a surface study was performed with **Fe-1** and **Fe-2** using a quartz crystal microbalance (QCM) and X-ray photoelectron spectroscopy (XPS); see the Supporting Information for full details. This study will allow better understanding of the orientation of these complexes in the molecular junction (single-molecule conductance properties and DFT studies) through the use of multipodal contacts. It is noteworthy that only **Fe-1** and **Fe-2** were used for this study since **Co-1** and **Co-2** complexes show practically the same molecular structure, and there is not a direct participation of the metal centers in the interaction with a gold substrate. Additionally, Fe(II) is the most electrochemically stable of the metals and, therefore, would provide the most reliable results.

To monitor the SAM formation from **Fe-1** and **Fe-2**, a QCM resonator was incubated in a 10^–4^ M solution in acetonitrile for each compound, and its frequency variation followed with respect to the incubation time. After 24 h, no further frequency variation was observed for either compound. This method gave a surface coverage of the resulting self-assembly monolayers as 1.1 × 10^–10^ and 1.2 × 10^–10^ mol·cm^–2^ for **Fe-1** and **Fe-2**, respectively, from the observed frequency variation of −14 Hz (**Fe-1**) and −31 Hz (**Fe-2**) and using the relationships described by the Sauerbrey equation.^19^ This shows that practically the same surface coverage (the same number of molecules per surface area) was obtained for both compounds, which suggests that **Fe-1** and **Fe-2** have the same orientation and arrangement in the SAM, even when **Fe-2** is much longer. This result suggests that the complexes assemble in a vertical orientation with respect to the gold substrate interacting through some of the six potential thiomethyls, giving the same effective area per molecule.

To obtain more details about the nature of the interaction between **Fe-1** and **Fe-2** and a gold substrate and hence gain information about the molecular orientation, XPS measurements were carried out on each of these iron compounds for both a powdered sample and SAMs on gold.

**Figure 3:**
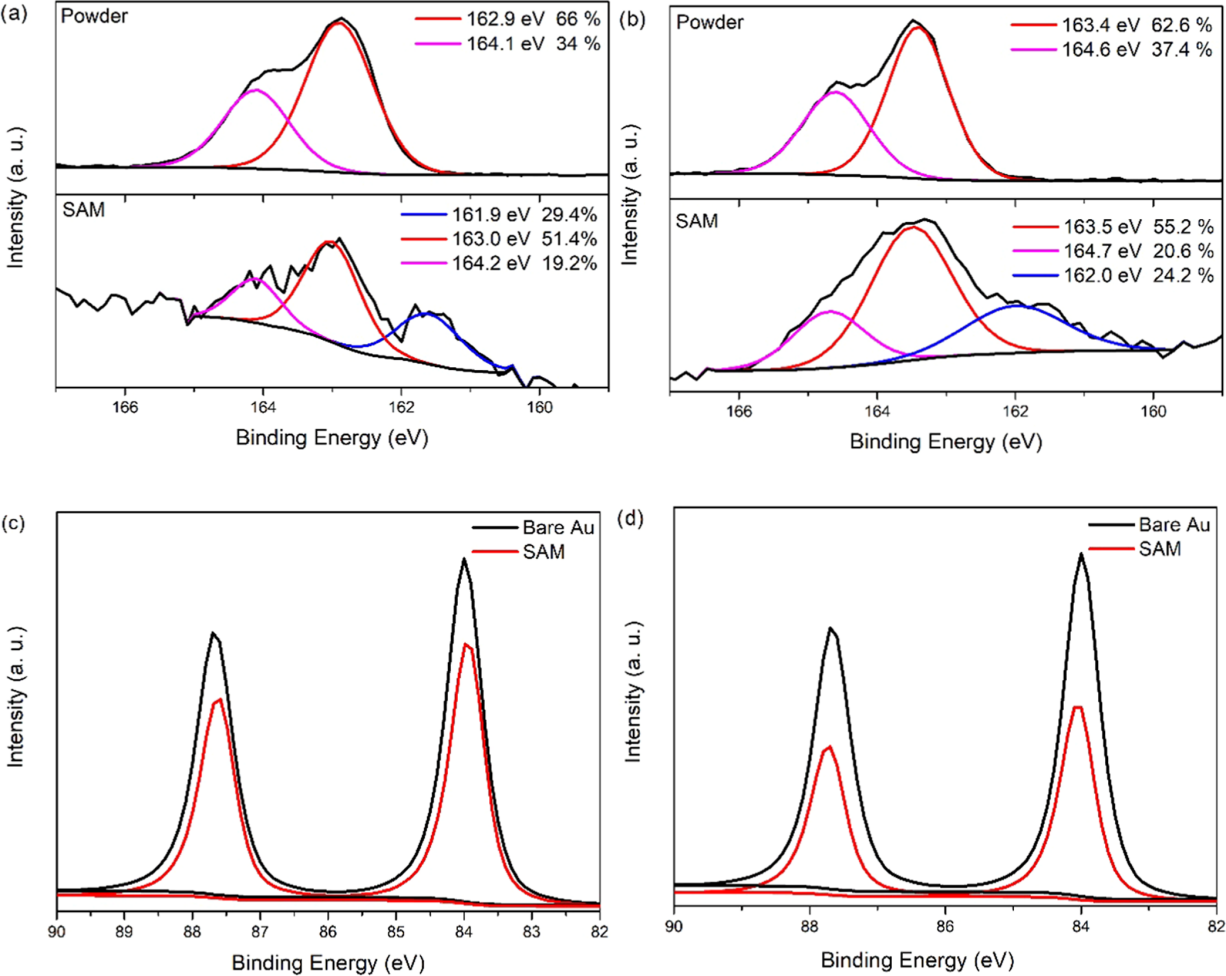
XPS spectra recorded for (a) **Fe-1** in the S 2p region in powder and SAM form on gold; (b) **Fe-2** in the S 2p region in powder and SAM form on gold; (c) **Fe-1** recorded in the Au 4f region for the uncovered Au substrate and the substrate covered by a SAM; and (d) **Fe-2** recorded in the Au 4f region for the uncovered Au substrate and the substrate covered by a SAM.

A powder sample of **Fe-1** in the S 2p region (Figure 3) of the XPS spectrum displayed two peaks at 162.9 and 164.1 eV (with a peak separation of 1.2 eV and an area ratio of 2:1 (66 and 34%)), assigned to (2p_3/2_) and (2p_1/2_), respectively. As expected, this shows that all sulfur atoms in the powder sample are in a chemically equivalent environment. In contrast, a SAM of **Fe-1** on a gold substrate gives a more complex XPS spectrum. The most intense pair of S 2p peaks at 163.0 and 164.2 eV is at practically the same binding energy as those observed for the powder sample. This indicates that at least some of the thiomethyls in **Fe-1** are free; in other words, they do not interact with the gold substrate. On the other hand, the peak at the lower binding energy (161.6 eV) was attributed to a S 2p_3/2_ peak, arising from thiomethyls interacting with the gold substrate.^7,20,21^ It would be expected that the corresponding weaker 2p_1/2_ peak would fall ca. 1.2 eV higher in energy (i.e., at 162.8 eV), but this is obscured by the more intense peaks from the thiomethyls that are not attached to the surface.

Therefore, the XPS data described above indicate that for a SAM of **Fe-1**, the molecules bind to the Au substrate through some of the potential thiomethyls contacting groups of **Fe-1**. Given that each S 2p_3/2,1/2_ doublet has a branching ratio of 2:1 (S 2p_3/2_/S 2p_1/2_), and taking into account the relative intensities of the clearly observed peaks at 164.1 eV (nonbound thiomethyls, S 2p_3/2_) and 161.4 eV (bound thiols, S 2p_1/2_), the corresponding areas associated with each signal in the convoluted spectrum can be estimated at: 19.2% (164.2 eV), ∼38% (163.0 eV), ∼14.5% (∼162.8 eV), and 29.4% (161.6 eV). From this, the relative area of XPS signals arising from thiomethyls not bound and bound to the gold substrate is approximated as 57:43. An estimate of the relative proportions of thiomethyls bound vs not bound was made, by using the attenuation of the Au 4f XPS signal (given the broadly similar binding energies of Au 4f and S 2p) from the gold substrate covered by a SAM of **Fe-1** (Figure 3). An attenuation factor of 0.76 was estimated. Using this attenuation factor, if three out of the six potential thiomethyl contacting groups were not bound and three were bound in the film of **Fe-1** on gold, this situation would be expected to give rise to XPS signals with an approximate ratio of 55:45. This ratio is very close to the integrated areas of the peaks observed in the experimental spectra (57:43).

The XPS data described above indicate that for the SAM of **Fe-1**, the molecules adsorb to the gold substrate through three of the six potential thiomethyl contacting groups; with the opposite three thiomethyls exposed on the top surface of the film and thereby available to contact a top electrode. In addition, the thickness of the SAM of **Fe-1** was also obtained through an analysis of the attenuation of the Au 4f signal in the XPS spectra (Figure 3), using the relationship *I*_SAM_ = *I*_substrate_ exp(−*d*/λ sin θ), where the film thickness is *d*; *I*_SAM_ and *I*_substrate_ are the combined average of the intensities of the Au 4f_5/2_ and Au 4f_7/2_ peaks from the SAM and from the bare gold, respectively; θ is the photoelectron take-off angle (90°); and λ is the effective attenuation length of the photoelectron (4.2 ± 0.1 nm).^22^ From this analysis, a thickness of 1.14 nm is obtained, which is consistent with having the molecules adsorbed to the gold substrate through three of the six potential thiomethyl contacting groups, as observed by XPS.

A similar study was carried out for a SAM of **Fe-2**. In this instance, the XPS spectrum for a powder sample of **Fe-2** in the S 2p region (Figure 3) showed two peaks at 163.4 and 164.6 eV (with a peak separation of 1.2 eV and an area ratio of 2:1 (63 and 37%)) assigned to (2p_3/2_) and (2p_1/2_), respectively. Meanwhile, a SAM of **Fe-2** on a gold substrate provided an XPS spectrum with the most intense pair of S 2p peaks, at 163.5 and 164.7 eV, practically at the same binding energy as those observed for the powder sample. These are associated with the thiomethyls not interacting with the gold substrate. A peak at a lower binding energy (162.0 eV) is attributed to the S 2p_3/2_ peak and arises from thiomethyls interacting with the gold substrate,^7,20,21^ with the corresponding weaker 2p_1/2_ peak being expected to fall ca. 1.2 eV higher in energy (i.e., at 163.2 eV) but obscured by the more intense peaks from the thiomethyls not attached to the surface. As mentioned above, given that each S 2p_3/2,1/2_ doublet has a branching ratio of 2:1 (S 2p_3/2_/S 2p_1/2_), and taking into account the relative intensities of the clearly observed peaks at 164.7 eV (nonbound thiolmethyls, S 2p_3/2_) and 162.0 eV (bound thiols, S 2p_1/2_), the corresponding areas associated with each signal in the convoluted spectrum can be estimated at: 20.6% (164.7 eV), ∼41% (163.5 eV), ∼12% (∼163.2 eV), and 24.2% (162.0 eV). That is, the relative area of XPS signals arising from thiomethyls not bound and bound to the gold substrate was approximately 64:36. Using the attenuation factor (0.56) of the Au 4f XPS signal from the gold substrate covered by a SAM of **Fe-1** (Figure 3), an estimate of the relative proportions of thiomethyls bound vs not bonded was made. Again, if three out of the six potential thiomethyl contacting groups were not bound and three were bound in the SAM of **Fe-2** on gold, this would give rise to XPS signals with an approximate ratio of 65:35 (i.e., very close to the integrated areas of the peaks observed in the experimental spectra). Therefore, these results indicate that for a SAM of **Fe-2**, the molecules adsorb to the gold substrate through three of the six potential thiomethyl contacting groups. The opposite three thiols are exposed on the top surface of the film and are thereby available to contact a top electrode, similar to compound **Fe-1**. Finally, using the attenuation of the Au 4f signal in the XPS spectra (Figure 3), a thickness of 2.4 nm was obtained, again consistent with SAM binding to the gold substrate through three of the six potential thiomethyl contacting groups. The XPS observations corroborate the QCM results; that a same surface coverage for **Fe-1** and **Fe-2** is indicative of a vertical orientation of the molecules with respect to the surface through three of the six potential thiomethyl groups.

### Conductance

**Figure 4:**
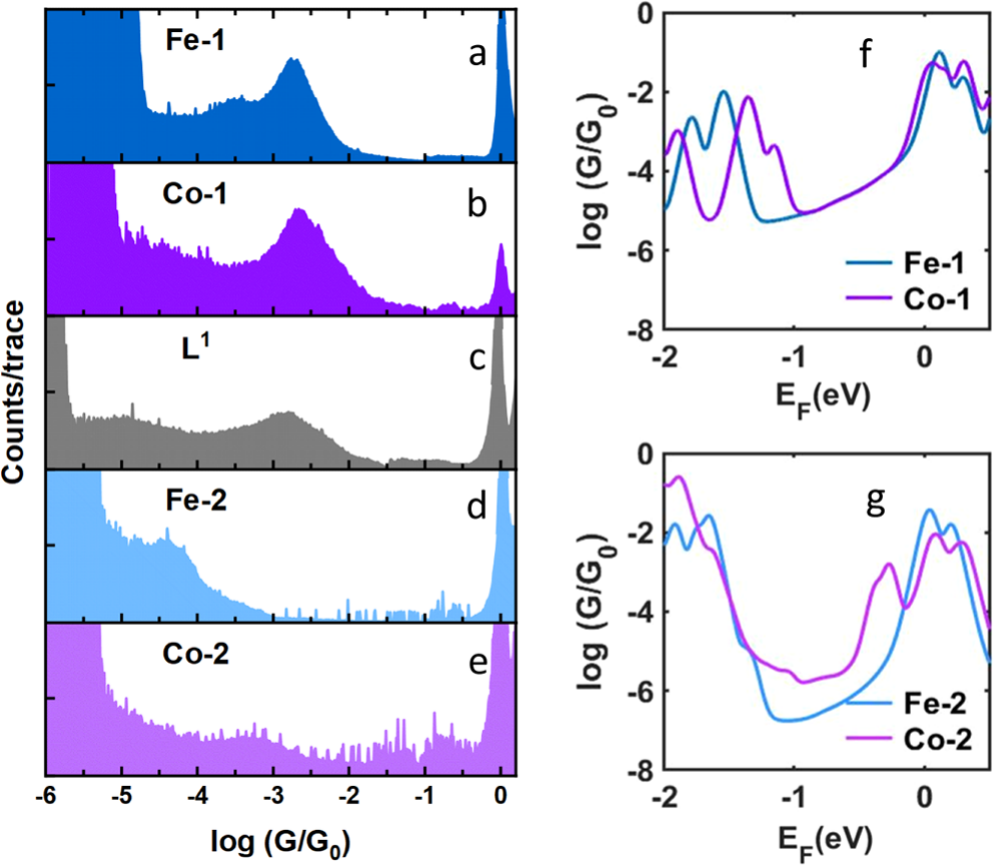
(a, b) 1-Dimensional conductance histograms of **Fe-1** (blue) and **Co-1** (purple). (c) Gray shows the conductance histogram of the ligand, **L**^**1**^, used in **Fe-1** and **Co-1**. Single-molecule conductance histograms indicate that the metal centers do participate in the dominant transport pathway. (d, e) Histograms of **Fe-2** (light blue) and **Co-2** (light purple). The right panels show DFT-based electrical conductance of the molecules with central atoms (f) **Fe-1** and **Co-1** and (g) **Fe-2** and **Co-2**, respectively. The DFT predicted Fermi energy *E*_F_ lies close to the LUMO resonance. The relaxed structures of the molecules and the junctions are shown in Figures S32, S34, S36, and S38 in the SI. In the case of cobalt, the calculation is spin-polarized and the average of the spin up and down are plotted.

The conductances of complexes **Fe-1** and **Co-1** were measured using the STM-BJ technique recorded in propylene carbonate, with a molecular concentration of 1 mM and using an Apiezon wax insulated tip. The example traces and 2D histograms are given in the SI. All values for the single-molecule conductance were obtained by fitting the corresponding 1D histograms with Gauss functions. The single-molecule conductance properties of the helical complexes (**Fe-1** and **Co-1**) indicate that the metal centers do not actively participate in the dominant transport channel. This observation can be seen by comparing the results from both **Fe-1** and **Co-1**; see Figure 4a,b. Here, the average conductance values are given by the peaks at 1 × 10^–2.77^*G*/*G*_0_ for **Fe-1** and 1 × 10^–2.65^*G*/*G*_0_ for **Co-1**. We measured the ligand **L**^**1**^ by itself to compare how the conductance might change if no metal center is present. The average conductance value for the ligand is 10^–2.88^*G*/*G*_0_, which confirms that the contribution of the metals in the complexes is minimal, see Figure 4c. Since the magnitude of the conductance is similar to that of the ligand, it means that if the metal ion participates in the charge transport pathway, it does not significantly affect the conductance. However, it is more likely that the metal ion does not participate in charge transport, as has been found for other systems where the metal is part of an optional electron pathway.^13^

The similarity of the conductance values for **Fe-1** and **Co-1** with that of the individual ligand **L**^**1**^ led us to hypothesize that the anchoring between molecule and electrode is through a single thioether contact on opposite ends of the molecule and through a single ligand (see schematic in Figure 1). Moreover, the experimental data confirm this hypothesis because of a well-defined molecular peak around 10^–3^*G*_0_ in the histograms for all three compounds (see Figure 4a–c). These findings are supported by our transmission calculations using density functional theory (DFT) SIESTA^23^ combined with the quantum transport code Gollum^24^ as shown in Figures 4f and S40. Any other configuration for the monometallic wires would result in a higher expected conductance value as shown in Figures S32–S35 in the SI.

The conductances of complexes **Fe-2** and **Co-2** were also measured using the STM-BJ technique. The poor solubility of these targets in nonpolar solvents meant that they could only be dissolved in higher-polar solvents. However, due to the increased lengths of the complexes and thus lowered conductance, a wax-coated tip and polar solvent could not be employed for STM-BJ measurements due to the conductance being below the leakage current threshold. To circumvent this, the complexes were preadsorbed on the surface from 1 mM solutions in ethanol for 10 min, rinsed and dried with nitrogen, and then measured in the nonpolar solvent 1,3,5-trimethylbenzene (TMB).

**Figure 5:**
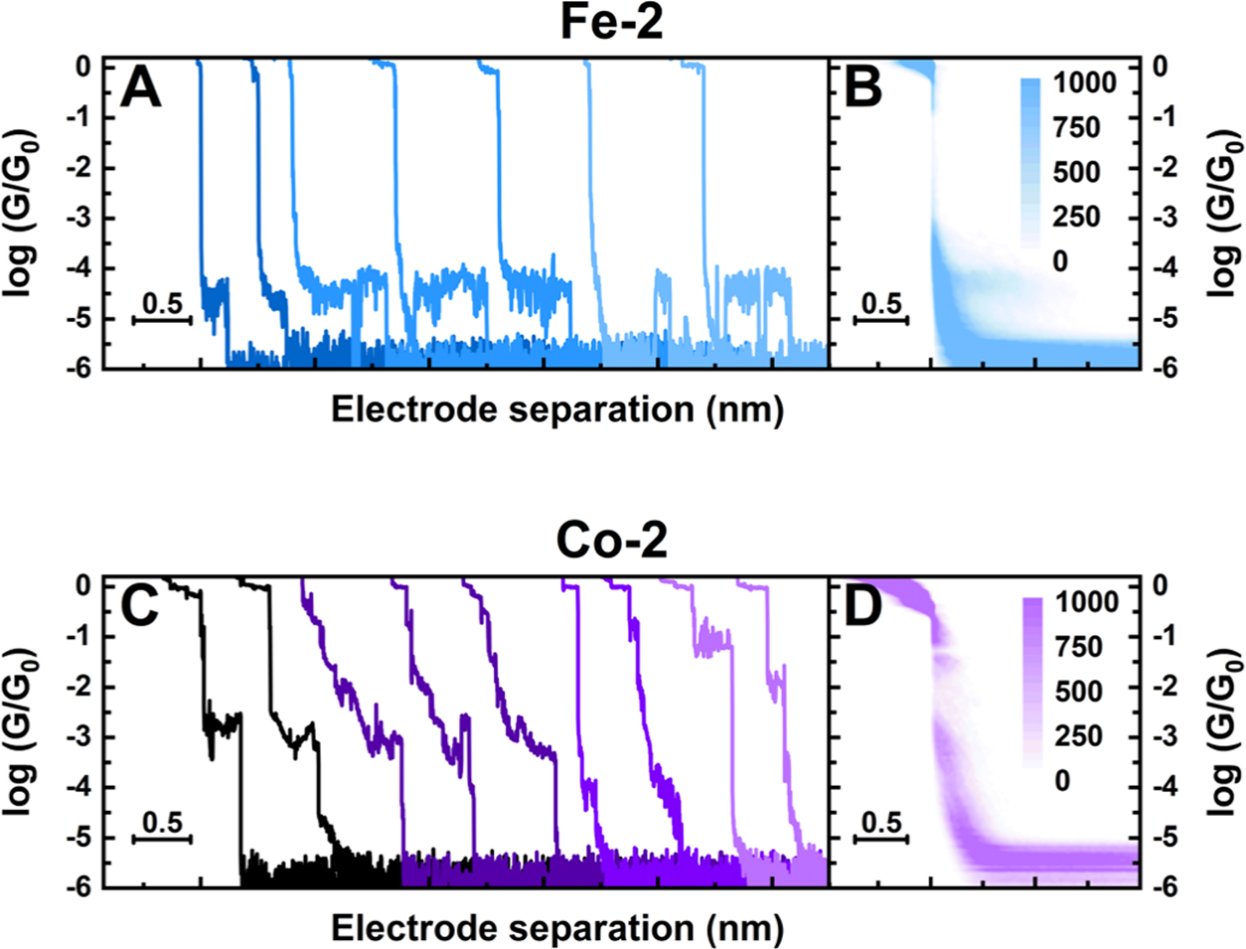
Example conductance-distance traces along with two-dimensional histograms. (A, B) Data set for **Fe-2** and (C, D) data set for **Co-2**.

The measurements of **Fe-2** and **Co-2** proved challenging, in contrast to the clear features of the shorter wires. Not only are the conductance values (expected to be) much lower but also the junction formation probability (the percentage of binding events) is limited. In fact, only the histogram for **Fe-2** shows a molecular feature at 10^–4.46^*G*/*G*_0_, see Figure 3d. We found that less than 10% of the traces for **Fe-2** contained a molecular plateau, indicating that the junction formation probability is low or that the junction is not that stable. Interestingly, about a quarter of these traces contained typical “blinking” events, see Figure 5a, which are routinely observed using current–time spectroscopy in which the tip–sample distance remains constant. One possibility here is that the electrode contacts one of the thioether anchoring groups and breaks off before contacting a second thioether group next to it. However, for the **Co-2** target, we observed molecular features at several different conductance values, see example traces in Figure 5c. Like **Fe-2**, only a small amount of the traces for **Co-2** do show a plateau, but its conductance values are spread over a large range. The first two example traces (in black) show a clearly defined plateau that corresponds to the faint feature that is present in the 2D histogram in Figure 5d. However, the next three example traces (in dark purple) show less well-defined features spanning across a wide conductance range. Yet other traces only show distinct features at low and high conductance values (two example traces in magenta and lilac, respectively). All of these outcomes combined make it challenging to interpret the exact behavior of **Co-2** in the junction.

### Theory

We performed unsupervised clustering analysis on the data sets for **Fe-2** and **Co-2**.^25^ Details of this process are provided in the SI. The algorithm separated the traces into three clusters; one cluster for **Co-2** and two clusters for **Fe-2** only contained clean tunneling (i.e., no evidence of junction formation). In the case of **Fe-2**, the remaining cluster, made of ∼26.5% of the data set, is characterized by the presence of plateaux having conductance values of ∼10^–4.5^*G*/*G*_0_ and extending to lengths commensurate to the theoretical S–S distance. These can be attributed to transport through an extended configuration of **Fe-2**. However, for **Co-2**, none of the clusters show formation of junctions that extend beyond a few Ångstroms, thereby our clustering analysis does not support the idea of transport through an extended conformation of the **Co-2** molecular wire. As the clustering algorithm identifies two different groups of traces, having conductances of ∼10^–1.5^ and 10^–3.3^*G*_0_, respectively, **Co-2** is either assembling in the electrode nanogap in a range of heavily tilted configurations or possibly decomposing in the junction.

The DFT calculations in Figure 4g (and Figures S36–S39 in the SI for other contact geometries) show a similar behavior for the conductance of **Fe-2** and **Co-2**. In general, the conductance of single-molecule compounds can vary depending on the contact configuration. However, the experimental data shows that the overall conductance for **Fe-2** is about 2 orders of magnitude lower than **Fe-1**. Comparing our experimental results with DFT calculations for single and multiple contacts (Figures S36 and S37 for **Fe-2** and Figures S38 and S39 for **Co-2**) suggests that such a large difference takes place when we only consider one connection to the electrodes. Also, the low percentage of junction formation probability confirms that it is less likely for the molecule to bind strongly to the gold electrode through multiple connections. Therefore, we conclude that the multiple contacts are less probable.

In the case of **Co-2**, conductance variations with different junction geometries are also impacted by the nature of the metal centers, since the magnetic properties of cobalt cause spin polarization, which creates an extra resonance close to Fermi energy due to different resonances in spin-up and -down transmission as shown in Figure S43 in the SI. This additional resonance leads to the molecular conductance being more sensitive to the precise junction geometry. Therefore, while we could expect similar charge transport efficiencies in the two bimetallic molecular wires, the failure of **Co-2** in adopting an extended configuration in the junction results in the observed very different conductance histograms.

## Conclusions

Four new metal complexes were synthesized, with each containing three independent molecular wires bound to metal centers to form a bundle. Through the use of QCM and XPS measurements, these complexes were shown to bind to a gold surface in a *fac* fashion through three thiomethyl groups, leaving three unbound. However, upon measuring the single-molecule conductance of the complexes, the only detected conductance paths originated from conductance through a single ligand, indicating that both the metal ion and the other ligands had little direct impact on the conductance of the ligand. This suggests that each of the ligands maintain their independent conductance behavior in the bundle, meaning that this approach can be used to control junction geometry without impacting the behavior of the individual conductive elements.

## Data Availability

Data collected using EPSRC funding at Liverpool are archived at 10.17638/datacat.liverpool.ac.uk/1992.

## Supporting Information Available

Details of synthetic procedures and characterization, NMR spectra, crystallographic data, electrochemical data, photochemical data, QCM details, XPS details, conductance details, example conductance trances, 2D histogram data, and details for theoretical calculations (PDF)

## References

[ref1] Su T. A.; Neupane M.; Steigerwald M. L.; Venkataraman L.; Nuckolls C. Chemical principles of single-molecule electronics. Nat. Rev. Mater. 2016, 1 (3), 16002 10.1038/natrevmats.2016.2.

[ref2] Nichols R. J.; Higgins S. J. Single molecule electrochemistry in nanoscale junctions. Curr. Opin. Electrochem. 2017, 4 (1), 98–104. 10.1016/j.coelec.2017.09.009.

[ref3] Tao N. Measurement and control of single molecule conductance. J. Mater. Chem. 2005, 15 (32), 3260–3263. 10.1039/b503307a.

[ref4] Wang K.; Xu B. Modulation and Control of Charge Transport Through Single-Molecule Junctions. Top. Curr. Chem. 2017, 375 (1), 17 10.1007/s41061-017-0105-z. 28120303

[ref5] Moore A. M.; Mantooth B. A.; Dameron A. A.; Donhauser Z. J.; Lewis P. A.; Smith R. K.; Fuchs D. J.; Weiss P. S. Frontiers in Materials Research; Fujikawa Y.; Nakajima K.; Sakurai T., Eds.; Springer Berlin Heidelberg: Berlin, Heidelberg, 2008; pp 29–47.

[ref6] O’Driscoll L. J.; Wang X.; Jay M.; Batsanov A. S.; Sadeghi H.; Lambert C. J.; Robinson B. J.; Bryce M. R. Carbazole-Based Tetrapodal Anchor Groups for Gold Surfaces: Synthesis and Conductance Properties. Angew. Chem., Int. Ed. 2020, 59 (2), 882–889. 10.1002/anie.201911652. PMC7027450 31714641

[ref7] Escorihuela E.; Cea P.; Bock S.; Milan D. C.; Naghibi S.; Osorio H. M.; Nichols R. J.; Low P. J.; Martin S. Towards the design of effective multipodal contacts for use in the construction of Langmuir–Blodgett films and molecular junctions. J. Mater. Chem. C 2020, 8 (2), 672–682. 10.1039/C9TC04710G.

[ref8] Šebera J.; Lindner M.; Gasior J.; Mészáros G.; Fuhr O.; Mayor M.; Valášek M.; Kolivoška V.; Hromadová M. Tuning the contact conductance of anchoring groups in single molecule junctions by molecular design. Nanoscale 2019, 11 (27), 12959–12964. 10.1039/C9NR04071D. 31259338

[ref9] Šebera J.; Kolivoška V.; Valášek M.; Gasior J.; Sokolová R.; Mészáros G.; Hong W.; Mayor M.; Hromadová M. Tuning Charge Transport Properties of Asymmetric Molecular Junctions. J. Phys. Chem. C 2017, 121 (23), 12885–12894. 10.1021/acs.jpcc.7b01105.

[ref10] Davidson R. J.; Milan D. C.; Al-Owaedi O. A.; Ismael A. K.; Nichols R. J.; Higgins S. J.; Lambert C. J.; Yufit D. S.; Beeby A. Conductance of ‘bare-bones’ tripodal molecular wires. RSC Adv. 2018, 8 (42), 23585–23590. 10.1039/C8RA01257A. 35540267 PMC9081744

[ref11] Ie Y.; Tanaka K.; Tashiro A.; Lee S. K.; Testai H. R.; Yamada R.; Tada H.; Aso Y. Thiophene-based Tripodal Anchor Units for Hole Transport in Single-Molecule Junctions with Gold Electrodes. J. Phys. Chem. Lett. 2015, 6 (18), 3754–3759. 10.1021/acs.jpclett.5b01662. 26722752

[ref12] Shen P.; Huang M.; Qian J.; Li J.; Ding S.; Zhou X.-S.; Xu B.; Zhao Z.; Tang B. Z. Achieving Efficient Multichannel Conductance in Through-Space Conjugated Single-Molecule Parallel Circuits. Angew. Chem., Int. Ed. 2020, 59 (11), 4581–4588. 10.1002/anie.202000061. 31943604

[ref13] Ponce J.; Arroyo C. R.; Tatay S.; Frisenda R.; Gavina P.; Aravena D.; Ruiz E.; van der Zant H. S. J.; Coronado E. Effect of Metal Complexation on the Conductance of Single-Molecular Wires Measured at Room Temperature. J. Am. Chem. Soc. 2014, 136 (23), 8314–8322. 10.1021/ja5012417. 24831452

[ref14] Komoto Y.; Yamazaki Y.; Tamaki Y.; Iwane M.; Nishino T.; Ishitani O.; Kiguchi M.; Fujii S. Ruthenium Tris-bipyridine Single-Molecule Junctions with Multiple Joint Configurations. Chem. - Asian J. 2018, 13 (10), 1297–1301. 10.1002/asia.201800166. 29528565

[ref15] Davidson R.; Al-Owaedi O. A.; Milan D. C.; Zeng Q.; Tory J.; Hartl F.; Higgins S. J.; Nichols R. J.; Lambert C. J.; Low P. J. Effects of Electrode–Molecule Binding and Junction Geometry on the Single-Molecule Conductance of bis-2,2′:6′,2″-Terpyridine-based Complexes. Inorg. Chem. 2016, 55 (6), 2691–2700. 10.1021/acs.inorgchem.5b02094. 26909823

[ref16] J Hannon M.; L Painting C.; Jackson A.; Hamblin J.; Errington W. An inexpensive approach to supramolecular architecture. Chem. Commun. 1997, (18), 1807–1808. 10.1039/A703713I.

[ref17] Gütlich P.; Goodwin H. A. Spin Crossover in Transition Metal Compounds I; Gütlich P.; Goodwin H. A., Eds.; Springer Berlin Heidelberg: Berlin, Heidelberg, 2004; pp 1–47.

[ref18] Glasson C. R. K.; Meehan G. V.; Davies M.; Motti C. A.; Clegg J. K.; Lindoy L. F. Post-Assembly Covalent Di- and Tetracapping of a Dinuclear [Fe2L3]4+ Triple Helicate and Two [Fe_4_L_6_]^8+^ Tetrahedra Using Sequential Reductive Aminations. Inorg. Chem. 2015, 54 (14), 6986–6992. 10.1021/acs.inorgchem.5b00940. 26125324

[ref19] Sauerbrey G. Verwendung von Schwingquarzen zur Wägung dünner Schichten und zur Mikrowägung. Z. Phys. 1959, 155 (2), 206–222. 10.1007/BF01337937.

[ref20] Sander F.; Hermes J. P.; Mayor M.; Hamoudi H.; Zharnikov M. Add a third hook: S-acetyl protected oligophenylene pyridine dithiols as advanced precursors for self-assembled monolayers. Phys. Chem. Chem. Phys. 2013, 15 (8), 2836–2846. 10.1039/c2cp43564k. 23337896

[ref21] Heister K.; Zharnikov M.; Grunze M.; Johansson L. S. O. Adsorption of Alkanethiols and Biphenylthiols on Au and Ag Substrates: A High-Resolution X-ray Photoelectron Spectroscopy Study. J. Phys. Chem. B 2001, 105 (19), 4058–4061. 10.1021/jp010127q.

[ref22] Bain C. D.; Whitesides G. M. Attenuation lengths of photoelectrons in hydrocarbon films. J. Phys. Chem. A 1989, 93 (4), 1670–1673. 10.1021/j100341a095.

[ref23] Soler J. M.; Artacho E.; Gale J. D.; Garcia A.; Junquera J.; Ordejon P.; Sanchez-Portal D. The SIESTA method for ab initio order-N materials simulation. J. Phys.: Condens. Matter 2002, 14 (11), 2745–2779. 10.1088/0953-8984/14/11/302.

[ref24] Ferrer J.; Lambert C. J.; Garcia-Suarez V. M.; Manrique D. Z.; Visontai D.; Oroszlany L.; Rodriguez-Ferradas R.; Grace I.; Bailey S. W. D.; Gillemot K.; Sadeghi H.; Algharagholy L. A. GOLLUM: a next-generation simulation tool for electron, thermal and spin transport. New J. Phys. 2014, 16, 093029 10.1088/1367-2630/16/9/093029.

[ref25] Cabosart D.; Abbassi M. E.; Stefani D.; Frisenda R.; Calame M.; van der Zant H. S. J.; Perrin M. L. A reference-free clustering method for the analysis of molecular break-junction measurements. Appl. Phys. Lett. 2019, 114 (14), 143102 10.1063/1.5089198.

