# Supplementary material for: Single-Molecule Conductance Behavior of Molecular Bundles
Source: Inorg Chem. 2023 Dec 11;62(51):20940–7. doi: 10.1021/acs.inorgchem.3c01943 (PMC10751792; doi:10.1021/acs.inorgchem.3c01943)
Supplement: Supplementary file 1 — ic3c01943_si_001.pdf [file ic3c01943_si_001.pdf]

SUPPORTING INFORMATION

Single Molecule Conductance Behaviour of Molecular Bundles

Alejandro Bara-Estaún,[a] Inco J. Planje,[b] Renad Almughathawi,[c,d] Saman Naghibi,[b] Andrea Vezzoli,[b] David C. Milan,[b] Colin Lambert [d], Santiago Martin,[e,f] Pilar Cea,[e,f] Richard J. Nichols,[b]* Simon J. Higgins,[b] Dmitry S. Yufit,[a] Sara Sangtarash,[g*] Ross J. Davidson,[a]* Andrew Beeby,[a]*.

[a]*Department of Chemistry, Durham University, South Rd, Durham, DH1 3LE, UK*

[b]*Department of Chemistry, University of Liverpool, Crown St, Liverpool, L69 7ZD, UK*

[c] *Department of Physics, Faculty of Science, Taibah University, Madinah, Saudi Arabia*

[d] *Department of Physics, University of Lancaster, Lancaster LA1 4YB, U.K*

[e] *Instituto de Nanociencia y Materiales de Aragón (INMA), CSIC-Universidad de Zaragoza, 50009, Zaragoza, Spain.*

[f] *Departamento de Química Física, Universidad de Zaragoza, 50009, Zaragoza, Spain and Laboratorio de Microscopias Avanzadas (LMA), Universidad de Zaragoza, 50018, Zaragoza, Spain.*

[g] *School of Engineering, University of Warwick, Coventry, U.K.*

Authors contributed equally.

*E-mail: R.J.Nichols@liverpool.ac.uk, sara.sangtarash@warwick.ac.uk, ross.davidson@durham.ac.uk and andrew.beeby@durham.ac.uk

Table of Contents

a) Experimental

1. Synthesis and characterisation of target compounds S3
2. NMR spectra of reported compounds S8
3. Crystallographic data S13
4. Electro-/photochemistry S15

b) Surface Characterisation

1. XPS. Experimental S25
2. QCM. Experimental S25

c) Conductance measurements

1. Conductance 1D and 2D Histograms S26

d) Theoretical details

1. Modelling charge transport through gold/metal-molecule/gold junctions S33
2. Computational methods S44

e) References S46

a) Experimental

1. Synthesis and characterisation of target compounds

General details. NMR spectra were recorded in deuterated solvent solutions on a Varian VNMRS-600 spectrometer and referenced against solvent resonances (^{1}H, ^{13}C). ASAP data were recorded using a Xevo QTOF (Waters) high resolution, accurate mass tandem mass spectrometer equipped with Atmospheric Pressure Gas Chromatography (APGC) and Atmospheric Solids Analysis Probe (ASAP). Microanalyses were performed by Elemental Microanalysis service, Durham University, UK. All chemicals were sourced from standard chemical suppliers, with the exception of 5,5'-dibromo-2,2'-bipyridine[1] and 2-bromo-5-(methylthio)pyridine[2] prepared following literature methods.

5,5'-bis(methylthio)-2,2'-bipyridine (L^1). n-BuLi (2.5 M, 1.6 mL, 4.00 mmol) was added to a solution containing 5,5'-dibromo-2,2'-bipyridine (500 mg, 1.60 mmol) in THF (50 mL) cooled to -78°C. The solution was stirred at -78 °C for 30 minutes before dimethyl sulphide (0.37 µL, 310 mg, 5.00 mmol) was added. The solution was allowed to warm to room temperature and stirring continued for an additional 16 hours. The reaction was quenched by the addition of water and extracted with dichloromethane, the organic layer was dried over $MgSO_4$ and the solvent removed. Hot methanol was added to the residue and filtered, the filtrate was collected and the solvent removed. The residue was recrystalised in hot hexane, upon cooling a white precipitate formed and was collected by filtration. **Yield**: 130 mg (33%). **^{1}H-NMR** ($CDCl_3$, 600 MHz) δ_H: 8.52 (d, $^{4}J_{HH}$ = 2.4 Hz, 2H), 8.27 (d, $^{3}J_{HH}$ = 8.3 Hz, 2H), 7.65 (dd, $^{3}J_{HH}$ = 8.3 Hz, $^{4}J_{HH}$ = 2.3 Hz, 2H), 2.54 (s, 6H) ppm. **^{13}C{^{1}H}-NMR** ($CDCl_3$, 126 MHz) δ_C: 152.5, 146.9, 135.5, 134.7, 120.4, 15.6 ppm. **MS(ESMS)**: m/z 249.1 $[M+H]^{+}$.

Anal. Calc. for $C_{12}H_{12}N_2S_2$: C, 58.03; H, 4.87; N, 11.28. **Found**: C, 58.06; H, 4.95; N, 11.07 %.

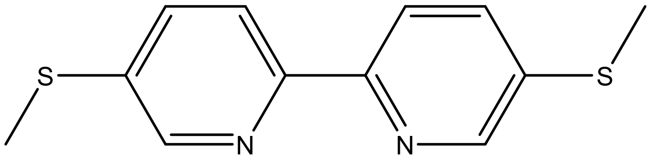

[Fe(L)$_3$](PF$_6$)(BF$_4$) (Fe-1). $Fe(BF_4)_2{\cdot}6H_2O$ (45 mg, 0.13 mmol) was added to a degassed solution of **L[1]** (100 mg, 0.40 mmol) in acetonitrile (30 mL) the solution was stirred at room temperature for 30 minutes before the solvent was removed. The red residue was dissolved in methanol (20 mL) and NH_4PF_6 (211 mg, 1.30 mmol) was added, the solvent was removed in vaccuo, the residue was dissolved in acetone and filtered. The solvent was removed and the residue was dissolved in acetonitrile and recrystallized via vapour diffusion of diethylether into the solution forming X-ray diffractable red crystals of **[Fe(L)$_3$](PF$_6$)$_2$**. **Yield**: 101 mg (72%). **^{1}H-NMR** (CD_3CN, 600 MHz) δ_H: 8.28 (dd, $^3J_{HH}$ = 8.7 Hz, $^4J_{HH}$ = 0.6Hz, 6H), 7.91 (dd, $^3J_{HH}$ = 8.7 Hz, $^4J_{HH}$ = 2.1 Hz, 6H), 7.03 (dd, $^3J_{HH}$ = 2.2 Hz, $^4J_{HH}$ = 0.6 Hz, 6H), 2.30 (s, 18H) ppm. **^{13}C{^{1}H}-NMR** (CD_3CN, 126 MHz) δ_C: 154.7, 149.2, 140.9, 134.7, 122.6, 13.5 ppm. **MS(ESMS)**: m/z 400.3 $[M\text{-}2PF_6]^{2+}$. **Anal. Calc.** for $C_{36}H_{36}BF_{10}FeN_6PS_6$: C, 41.87; H, 3.51; N, 8.14 %. **Found**: C, 41.77; H, 3.53; N, 8.02 %.

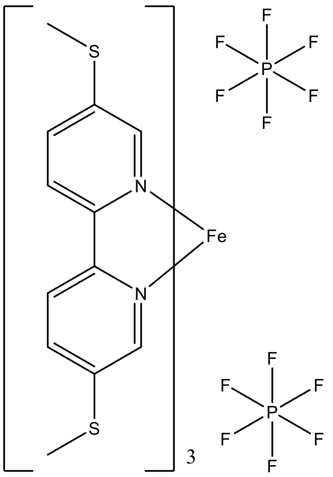

[Co(L)$_3$](PF$_6$)$_2$ (Co-1). Co(BF$_4$)$_2$·6H$_2$O (44 mg, 0.13 mmol) was added to a degassed solution of **L[1]** (100 mg, 0.40 mmol) in acetonitrile (30 mL) the solution was stirred at room temperature for 30 minutes before the solvent was removed. The red residue was dissolved in methanol (20 mL) and NH$_4$PF$_6$ (211 mg, 1.30 mmol) was added, the solvent was removed in vaccuo, the residue was dissolved in acetone and filtered. The solvent was removed and the residue dissolved in acetonitrile and recrystallized via vapour diffusion of diethylether into the solution forming X-ray diffractable red crystals. **Yield**: 77 mg (55%). **MS(ESMS)**: m/z 267.8 [M-2PF$_6$]$^{3+}$. **Anal. Calc.** for C$_{36}$H$_{36}$CoF$_{12}$N$_6$P$_2$S$_6$·C$_4$H$_{10}$O: C, 41.13; H, 3.97; N, 7.19 %. **Found**: C, 40.96; H, 3.96; N, 7.00 %.

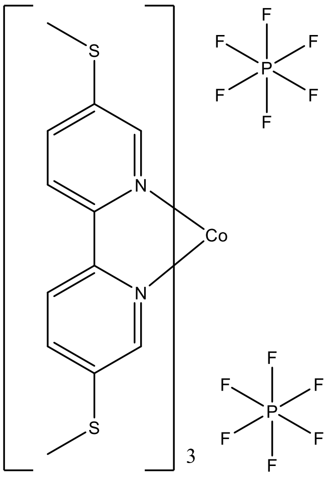

5-(methylthio)picolinaldehyde. t-BuLi (2.57 mL, 4.90 mmol, 1.9 M) was added slowly to a solution containing 2-bromo-5-(methylthio)pyridine (1.00 g, 4.90 mmol) in diethylether (50 mL) at -78 °C. The solution was stirred for 1 hour before DMF (0.39 mL, 5.00 mmol) was added, the solution was stirred for 30 minutes before being warmed to room temperature. The reaction was quenched with $NH_4Cl_{(aq)}$ and extracted with diethylether. The compound was purified by via silica chromatography eluted by a solvent gradient of DCM to acetone, after removal of solvent a yellow oil was obtained. **Yield**: 0.50 g (66 %). **^{1}H-NMR** ($CDCl_3$, 600 MHz) δ: 9.82 (d, $^4J_{HH}$ = 1.1 Hz, 1H, H_a), 8.39 (d, $^4J_{HH}$ = 2.4 Hz, 1H, H_b), 7.66 (d, $^3J_{HH}$ = 8.3 Hz, 1H, H_c), 7.45 (dd, $^3J_{HH}$ = 8.3 Hz, $^4J_{HH}$ = 1.0 Hz, 1H, H_d), 2.41 (s, 3H, H_e) ppm. **^{13}C{^{1}H}-NMR** ($CDCl_3$, 126 MHz) δ: 192.3, 148.9, 146.3, 142.5, 132.3, 14.2 ppm. **MS(ASAP)**: *m/z* 154.030 $[M+H]^+$.

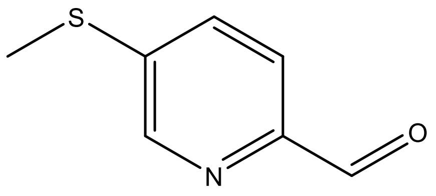

$[Fe_2L_3](BF_4)_4$ (Fe-2). 5-(methylthio)picolinaldehyde (155 mg, 2.0 mmol) was added to a solution containing 4,4'-methylenedianiline (200 mg, 1.0 mmol) in MeOH (40 mL). The solution was stirred at room temperature for 4 hours before $Fe(BF_4)_2{\cdot}6H_2O$ (224 mg, 0.66 mmol) was added to the suspension, this was heated to reflux for 4 hours before the solvent was removed. The purple residue was dissolved in acetonitrile and filtered through celite, diethylether was vapour diffused into the filtrate to give purple X-ray diffractable crystals. **Yield**: 498 mg (81%). **^{1}H-NMR** (CD_3CN, 600 MHz, 50 °C) δ: 9.38 (s, 6H), 8.55 (d, $^3J_{HH}$ = 8.4 Hz, 6H), 8.13 (d, $^3J_{HH}$ = 8.4 Hz, 6H), 7.49 (s, 6H), 6.97 (d, $^3J_{HH}$ = 7.3 Hz, 12 H), 5.57 (d,

$^{3}J_{HH}$ = 7.9 Hz, 12 H), 4.10 (s, 6H), 2.45 (s, 18H) ppm. **^{13}C{^{1}H}-NMR** (CD_3CN, 126 MHz, 25°C) δ:173.3, 153.4, 151.2, 149.5, 145.7, 141.5, 134.4, 129.9, 129.8, 121.8, 39.1, 13.5 ppm. **MS(ESMS)**: m/z 379.39 $[M\text{-}4BF_4]^{4+}$. **Anal. Calc.** for $C_{81}H_{72}B_4F_{16}Fe_2N_{12}S_6{\cdot}2H_2O$: C, 51.18; H, 4.03; N, 8.84 %. **Found**: C, 51.10; H, 3.73; N, 8.80 %.

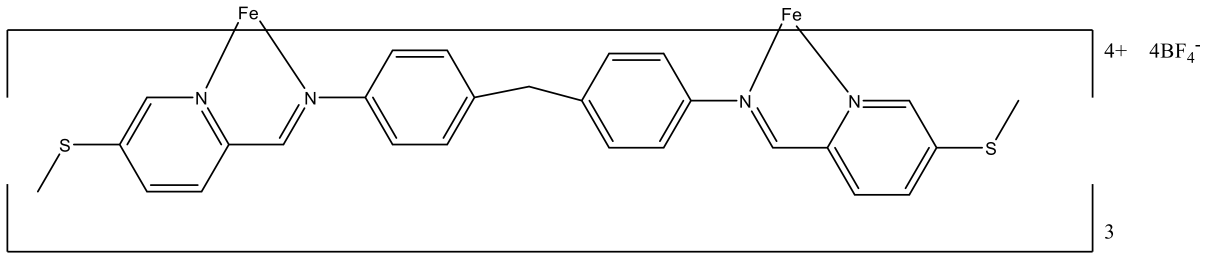

$[Co_2L_3](BF_4)_4$ (Co-2). The same procedure as for $[Fe_2L_3](BF_4)_4$ except $Co(BF_4)_2{\cdot}6H_2O$ was used in place of $Fe(BF_4)_2{\cdot}6H_2O$ to give red crystals. **Yield**: 423 mg (68%). **MS(ESMS)**: m/z 381.1 $[M\text{-}4BF_4]^{4+}$. **Anal. Calc.** for $C_{81}H_{72}B_4F_{16}Co_2N_{12}S_6{\cdot}H_2O$: C, 51.50; H, 3.95; N, 8.90 %. **Found**: C, 51.32; H, 3.78; N, 8.90 %.

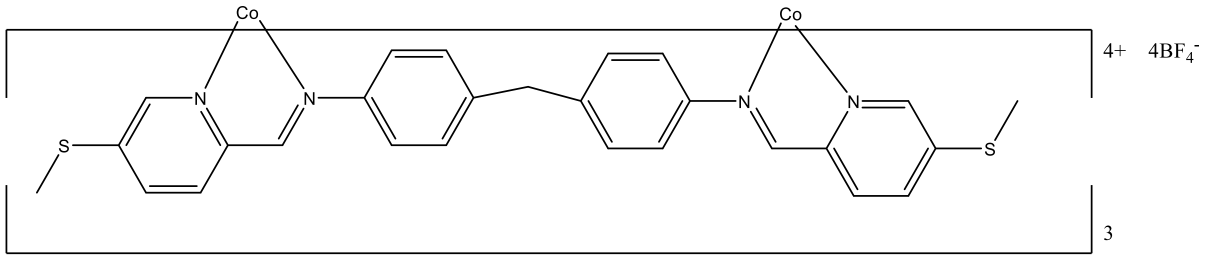

2. NMR spectra of reported compounds

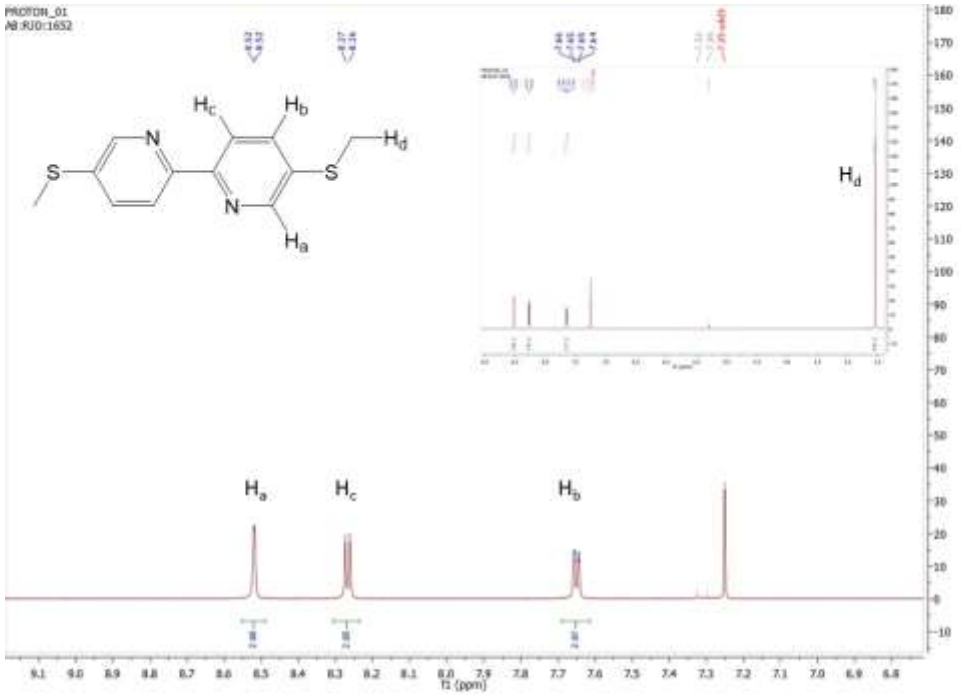

Figure S1. ^{1}H NMR spectrum of 5,5'-bis(methylthio)-2,2'-bipyridine (**L[1]**) recorded in $CDCl_3$.

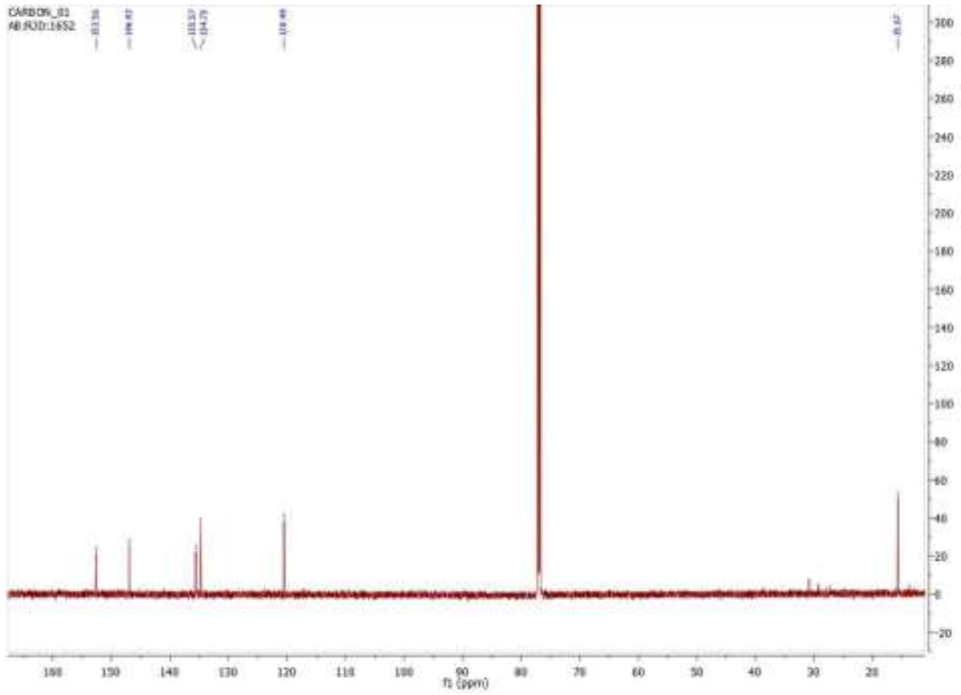

Figure S2. $^{13}C\{^1H\}$ NMR spectrum of 5,5'-bis(methylthio)-2,2'-bipyridine (**L[1]**) recorded in $CDCl_3$.

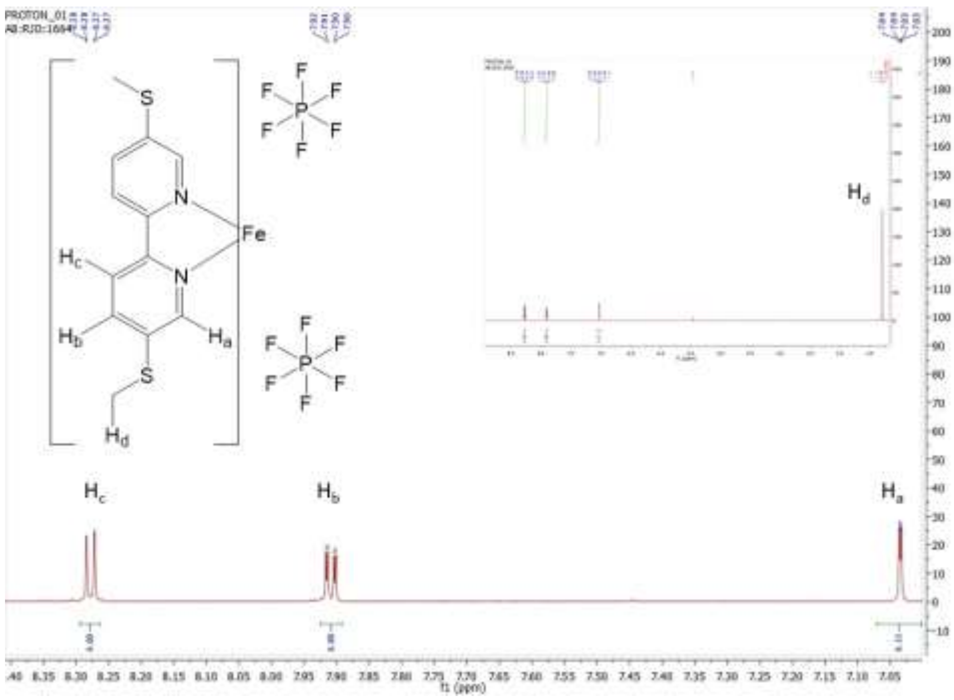

Figure S3. ^{1}H NMR spectrum of $[Fe(L)_3](PF_6)_2$ (**Fe-1**) recorded in CD_3CN.

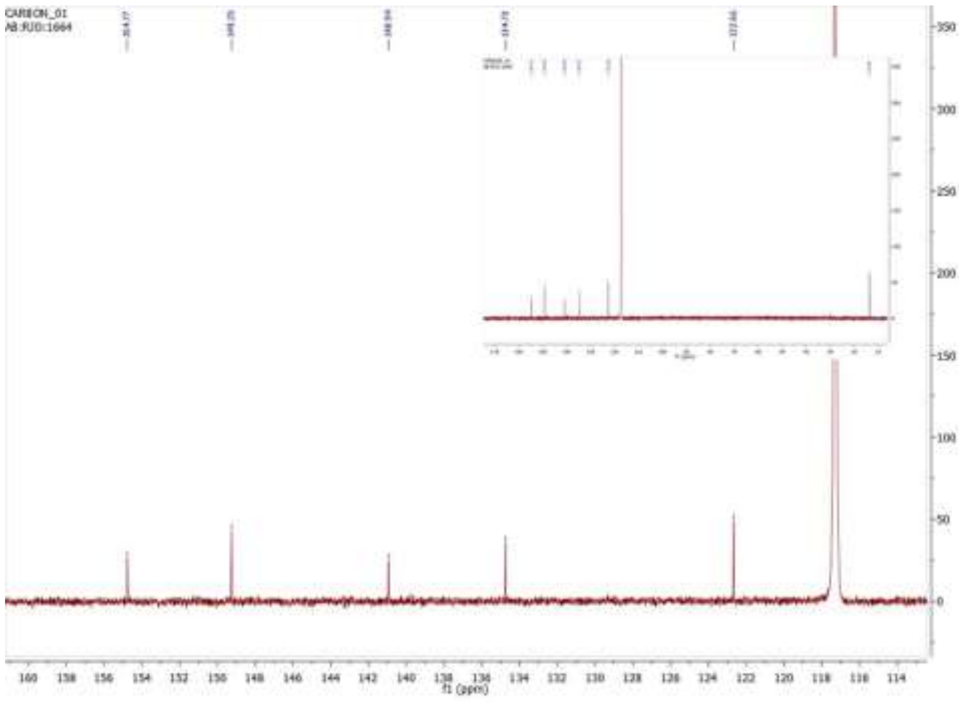

Figure S4. $^{13}C\{^{1}H\}$ NMR spectrum of $[Fe(L)_3](PF_6)_2$ (**Fe-1**) recorded in CD_3CN.

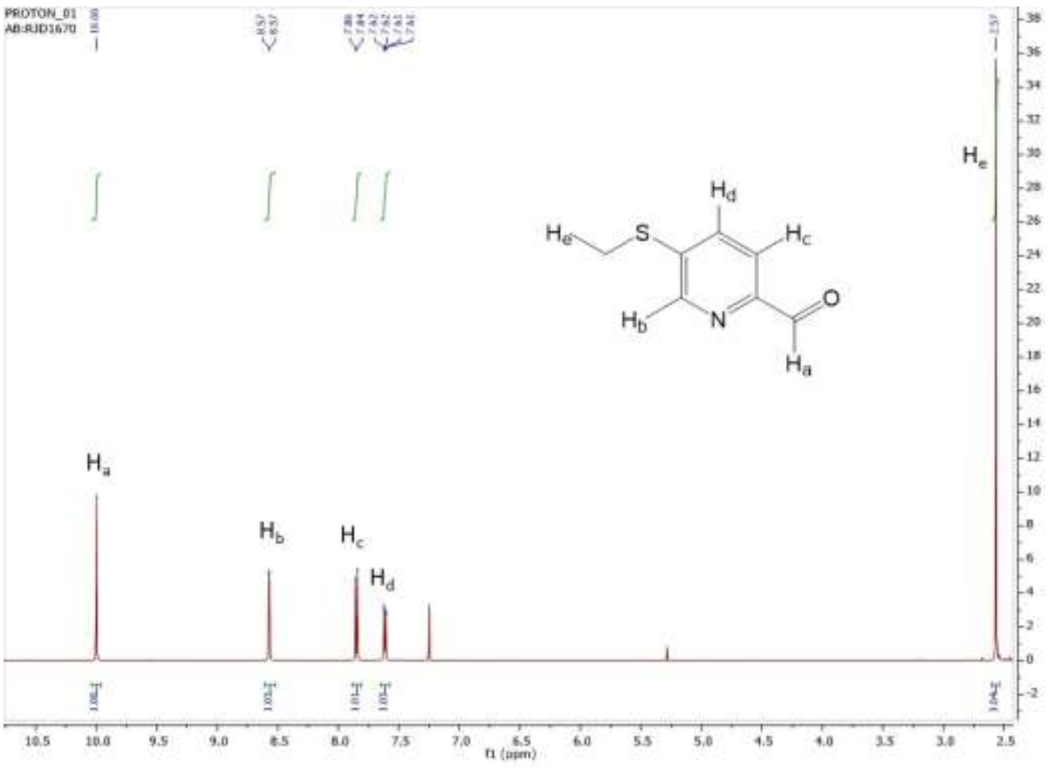

Figure S5. ^{1}H NMR spectrum of **5-(methylthio)picolinaldehyde** recorded in $CDCl_3$.

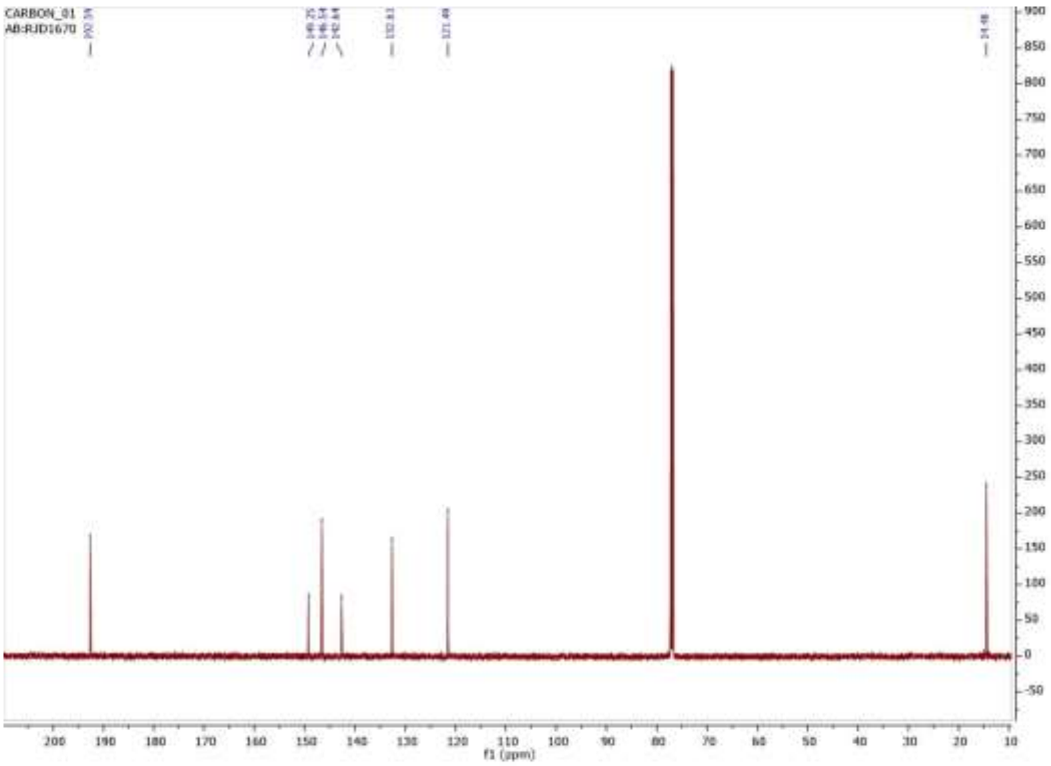

Figure S6. $^{13}C\{^{1}H\}$ NMR spectrum of **5-(methylthio)picolinaldehyde** recorded in $CDCl_3$.

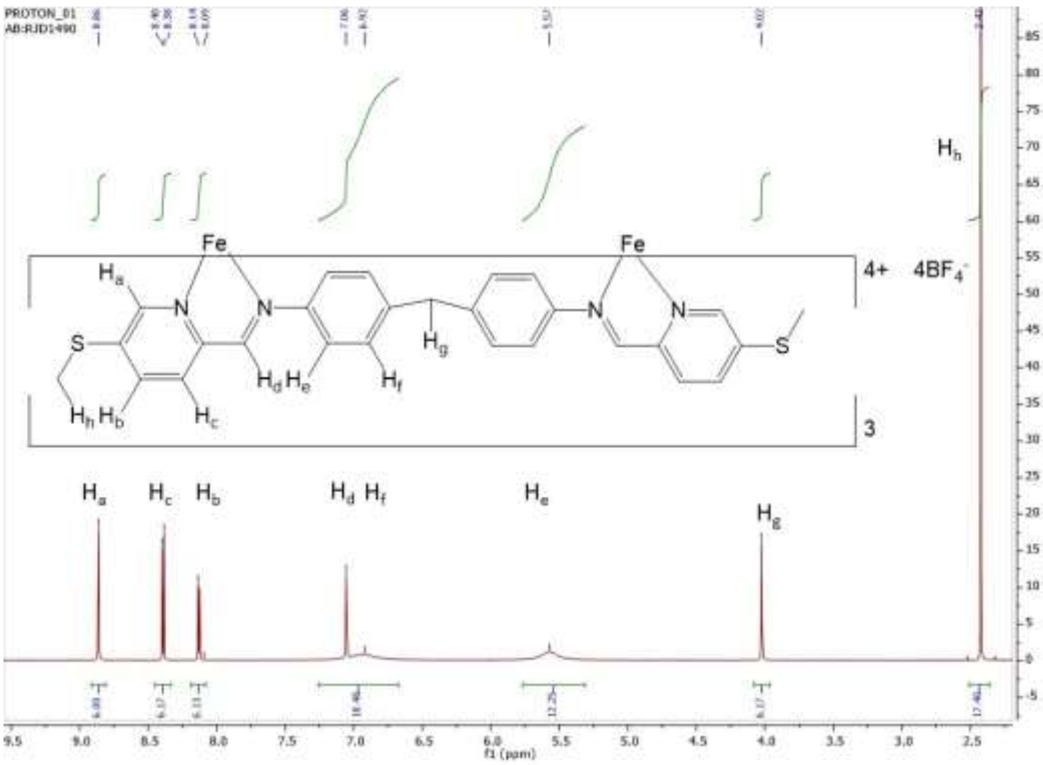

Figure S7. ^{1}H NMR spectrum of $[Fe_2L^2{}_3](BF_4)_4$ **(Fe-2)** recorded in CD_3CN at 25 °C.

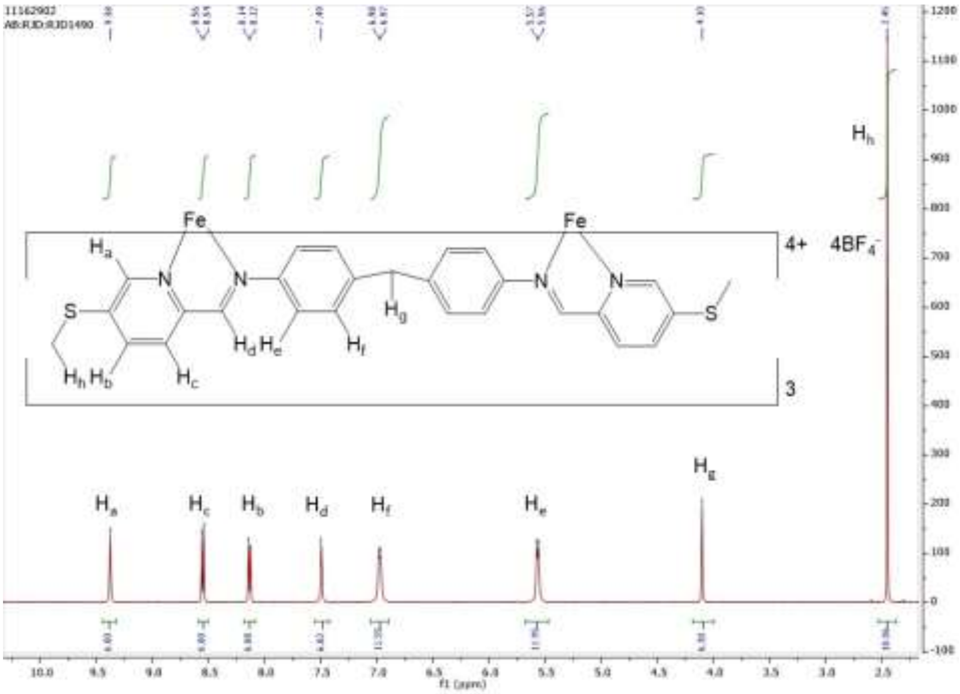

Figure S8. ^{1}H NMR spectrum of $[Fe_2L^2{}_3](BF_4)_4$ (**Fe-2**) recorded in CD_3CN at 50 °C.

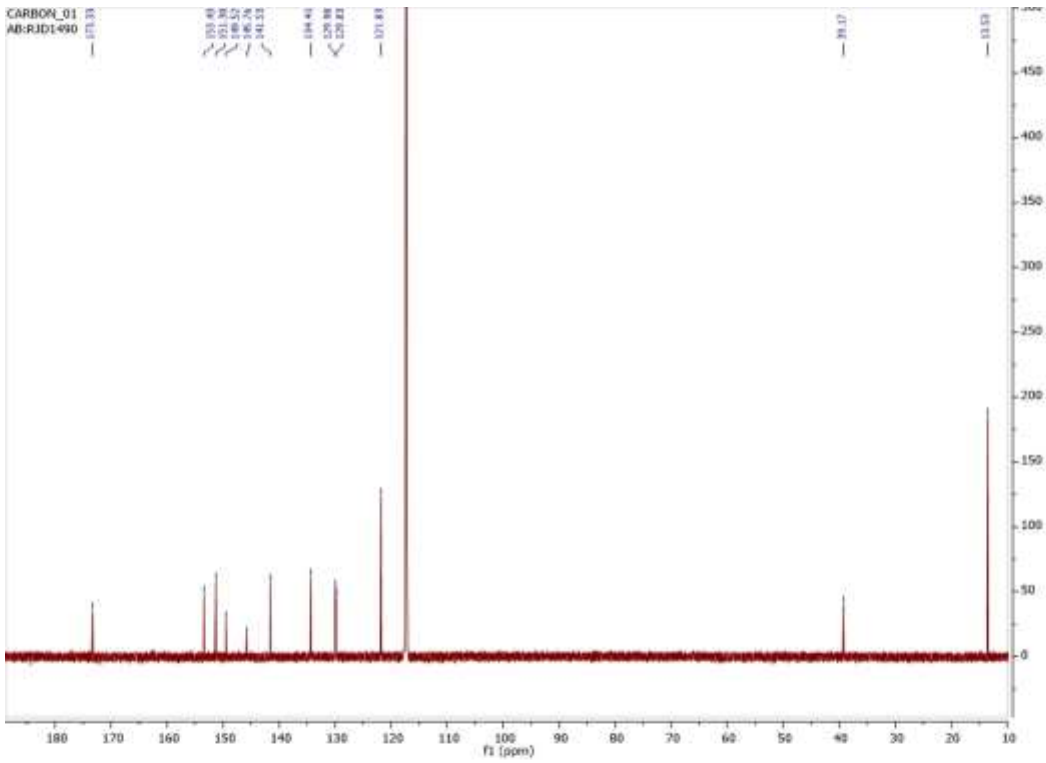

Figure S9. $^{13}C\{^1H\}$ NMR spectrum of $[Fe_2L^2_3](BF_4)_4$ (**Fe-2**) recorded in CD_3CN at 25 °C.

3. Crystallographic data

The X-ray single crystal data have been collected at the temperature 120.0(2) K (220.0(2) K for **Co-2**) using λMoKα radiation (λ =0.71073Å) on a Bruker D8Venture (compounds **Fe-1** a, **Co-1** and **Co-2**; Photon100 CMOS detector, IμS-microsource, focusing mirrors) and an Agilent XCalibur (compounds **Fe-2**; Sapphire-3 CCD detector, fine-focus sealed tube, graphite monochromator) diffractometers equipped with Cryostream (Oxford Cryosystems) open-flow nitrogen cryostats. All structures were solved by direct method and refined by full-matrix least squares on F^2 for all data using Olex2[3] and SHELXTL[4] software. All non-disordered non-hydrogen atoms were refined anisotropically, the hydrogen atoms were placed in the calculated positions and refined in riding mode. Disordered atoms in all structures were refined isotropically with fixed SOF=0.5. One of the severely disordered acetonitrile solvent molecules in the structure **Fe-1** could not be reliably modelled and has been taken into consideration using the MASK procedure of the OLEX2 package (appr. 24 independent electrons). Similarly, 5 independent acetonitrile molecules (107 e) were MASKed in the structure **Co-2**. Crystal data and parameters of refinement are listed in Table S1. Crystallographic data for the structure have been deposited with the Cambridge Crystallographic Data Centre as supplementary publication CCDC 2245744-2245747 .

Table S1. Crystal data and structure refinement for structures **Fe-1**, **Co-1**, **Fe-2**, and **Co-2**.

Identification code	**Fe-1**	**Co-1**	**Fe-2**	**Co-2**
Empirical formula	$C_{36}H_{36}FeN_6S_6 \times 2PF_6 \times 3CH_3CN$	$C_{36}H_{36}CoN_6S_6 \times 2PF_6 \times CH_3CN$	$[C_{81}H_{72}Fe_2N_{12}S_6]^{4+} \times 4[BF_4]^- \times 8\,CH_3CN$	$[C_{81}H_{72}Co_2N_{12}S_6]^{4+} \times 4[BF_4]^-$
Formula weight	1214.02	1134.99	2193.23	1869.86
Crystal system	monoclinic	monoclinic	monoclinic	monoclinic
Space group	C2/c	C2/c	C2/c	C2/c
a/Å	27.1182(19)	17.7059(15)	22.1297(5)	21.3482(10)
b/Å	15.1995(11)	17.9269(15)	12.9165(3)	14.0533(6)
c/Å	28.774(3)	16.8095(14)	36.2783(9)	36.9590(16)
β/°	110.431(2)	111.457(3)	94.153(2)	93.848(2)
Volume/Å^3	11114.0(16)	4965.7(7)	10342.5(4)	11063.2(8)
Z	8	4	4	4
$\rho_{calc,}$ g/cm^3	1.451	1.518	1.409	1.123
μ/mm^{-1}	0.634	0.744	0.487	0.480
F(000)	4960.0	2308.0	4520.0	3820.0
Reflections collected	102139	50823	58951	92254
Independent refl., R_{int}	14768 0.0546	6585 , 0.0391	11307, 0.1095	13352, 0.1186
Data/restraints/parameters	14768/195/651	6585/2/308	11307/40/648	13352/49/547
Goodness-of-fit on F^2	1.040	1.035	1.043	1.052
Final R_1 indexes [I≥2σ (I)]	0.0822	0.0512	0.0764	0.0788
Final wR_2 indexes [all data]	0.2219	0.1553	0.2213	0.2275

4. Electro-/photochemistry

Cyclic voltammograms were recorded for mononuclear complexes in 0.1 M $TBAPF_6$ (complexes **Fe-1** and **Co-1**) or $TBABF_4$ (complexes **Fe-2** and **Co-2**) in acetonitrile, and referenced against ferrocene (i.e. $E_{½}$ $FeCp_2$ / $[FeCp_2]^+$ = 0.00 V), see table S2 for tabulated data. Both **Fe-1** and **Fe-2** show only a single oxidation wave at 0.72 and 0.82 V, respectively, attributed to the Fe(II)/Fe(III) couple. **Co-1** and **Co-2** both display a metal centred oxidation wave -0.10 and -0.09V respectively attributed to the Co(II)/Co(III) couple with two further ligand based reductions. Additionally, to examine if any through space interactions were possible with these systems, $TBArF_{24}$ was used as the electrolyte. Vincent *et al*. has previously demonstrated that the use of such a large anion typically increases the separation of oxidation events linked by through-space interactions.[5, 6] However, for **Fe-2** and **Co-2** only a single oxidation event was observed, confirming the absence of electronic coupling between the metal centres.

Table S2. Electrochemical data for the complexes **Fe-1**, **Co-1**, **Fe-2**, and **Co-2**

**Compound**	**$E_{½}$($V_{Fc/Fc+}$)**	
	Oxidation	Reduction
$[Fe(bpy)_3](ClO_4)_2$[7]	0.66	1.75
$[Co(bpy)_3](ClO_4)_2$[7]	0.06	-1.35
**Fe-1**	0.72	-1.60[i]
**Co-1**	-0.10	-1.29, -1.96
**Fe-2**	0.82	
**Co-2**	-0.09	-1.15, -1.73[ii]

[i]Determined by differential pulse, [ii]irreversible reduction

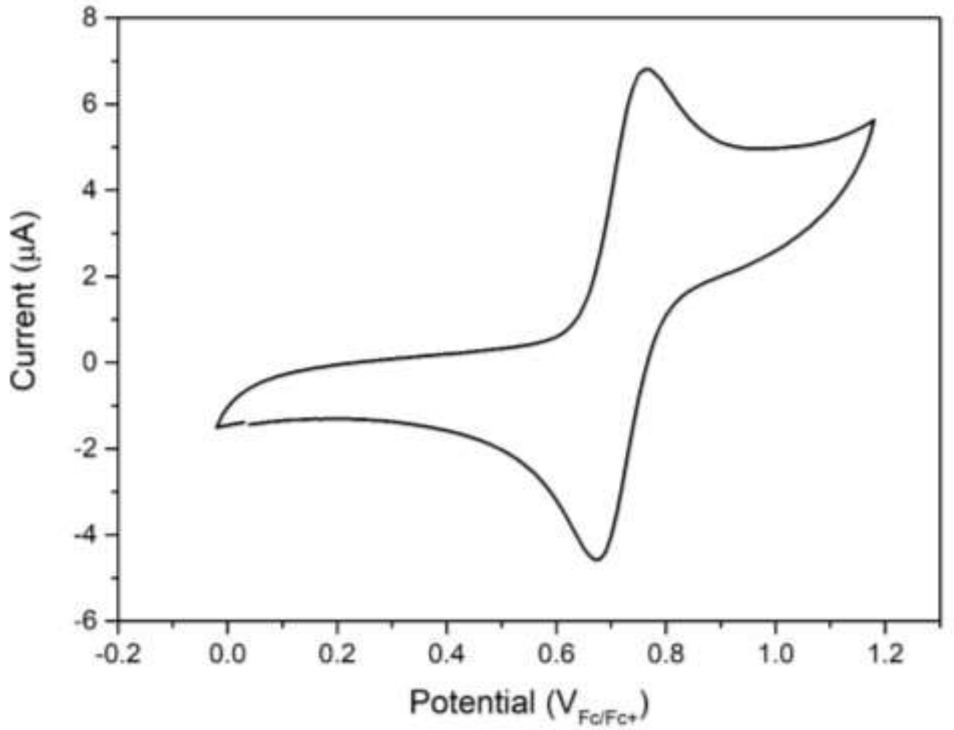

Figure S10. Cyclic voltammogram of complex **Fe-1** recorded with scan rate of 100 mV s^{-1}.

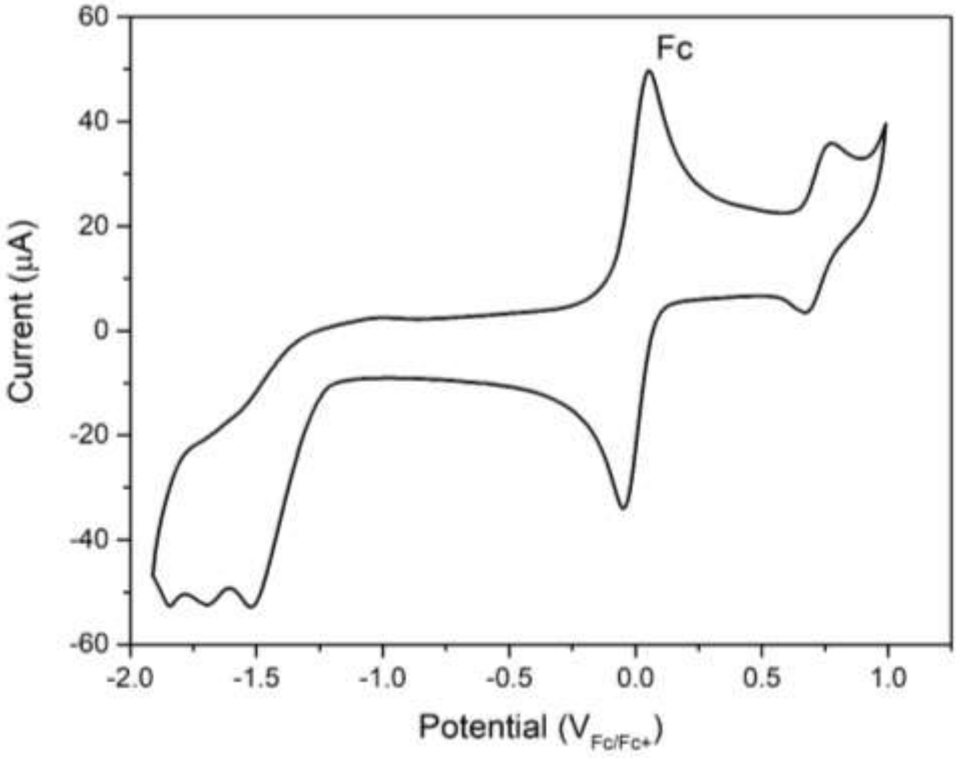

Figure S11. Cyclic voltammogram of complex **Fe-1** recorded with scan rate of 100 mV s^{-1}.

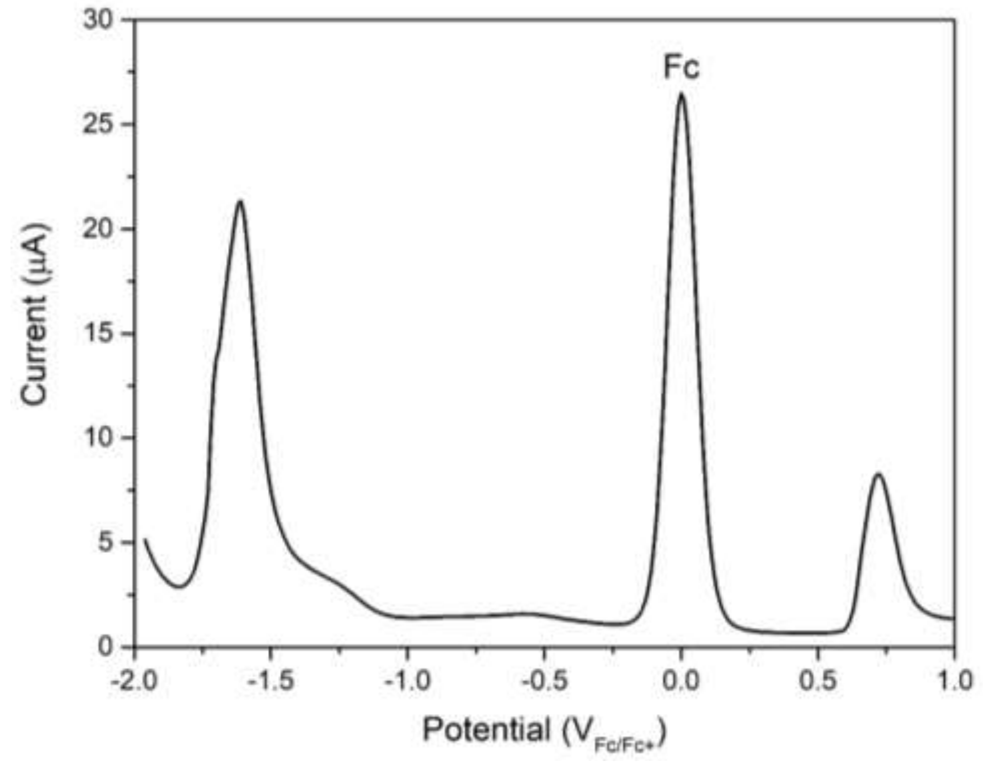

Figure S12. Differential pulse voltammogram of complex **Fe-1**.

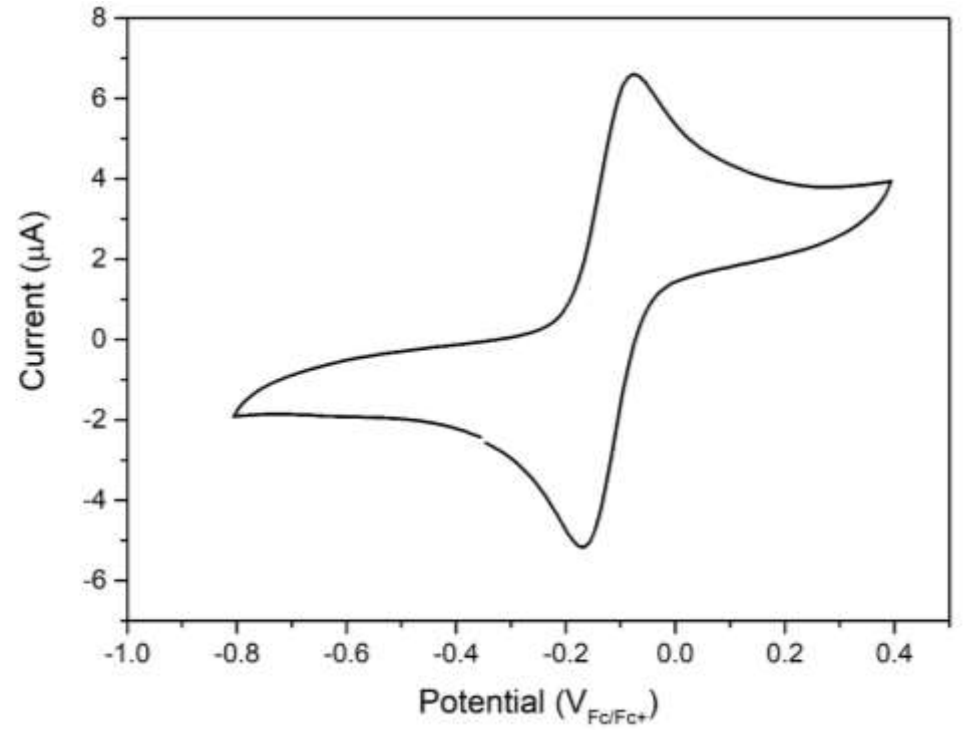

Figure S13. Cyclic voltammogram of complex **Co-1** recorded with scan rate of 100 mV s^{-1}.

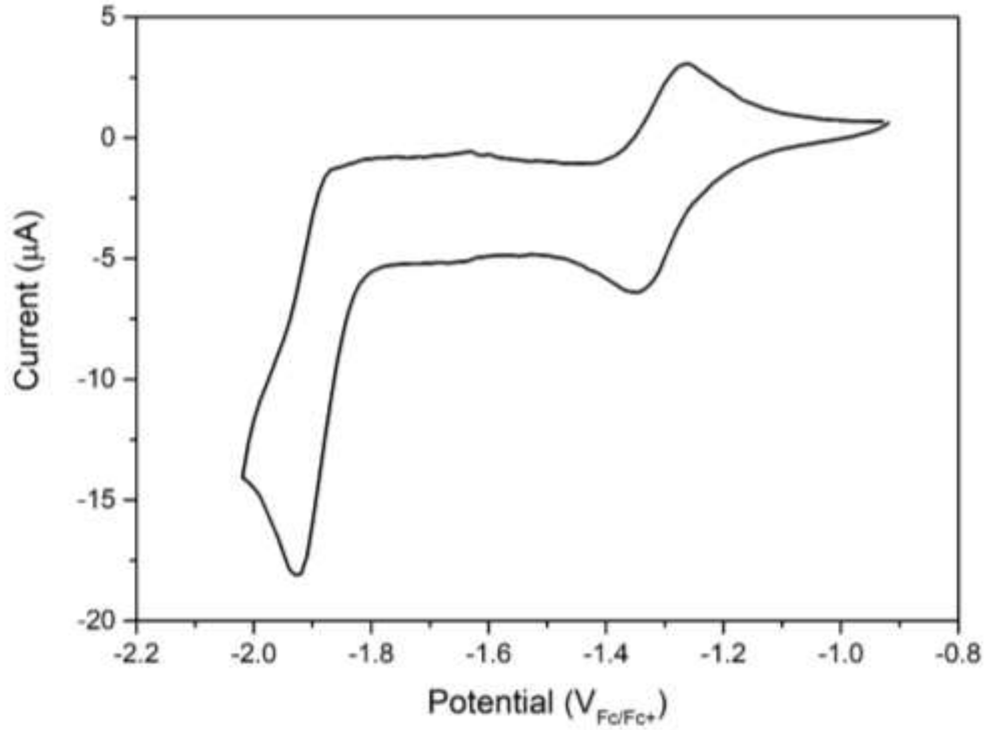

Figure S14. Cyclic voltammogram of complex **Co-1** recorded with scan rate of 100 mV s^{-1}.

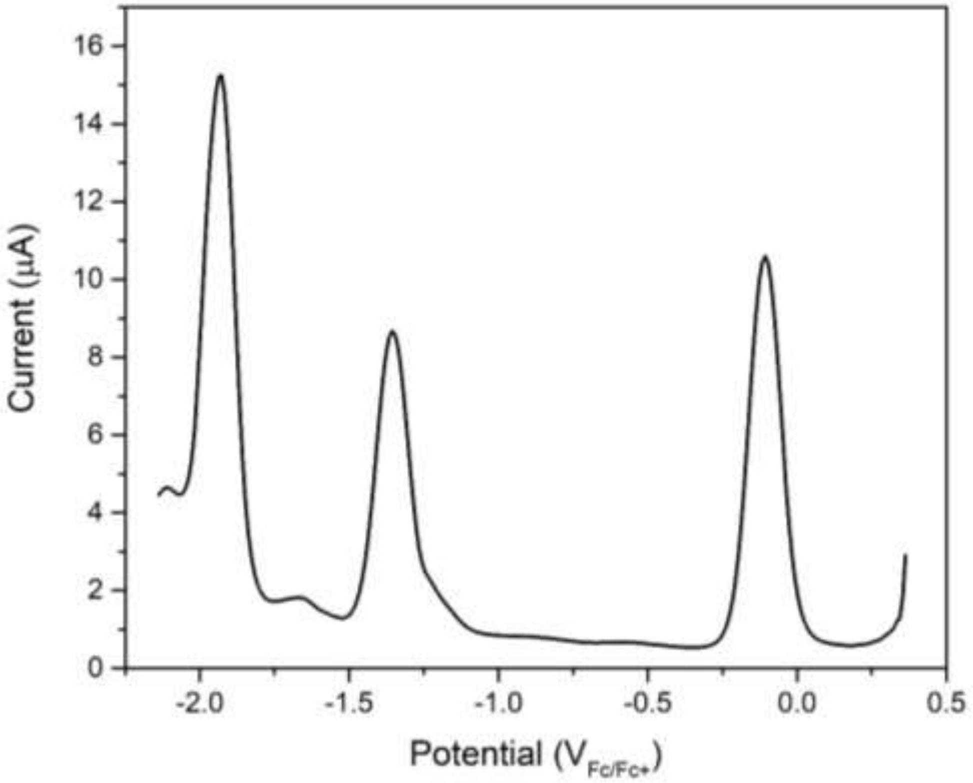

Figure S15. Differential pulse voltammogram of complex **Co-1**.

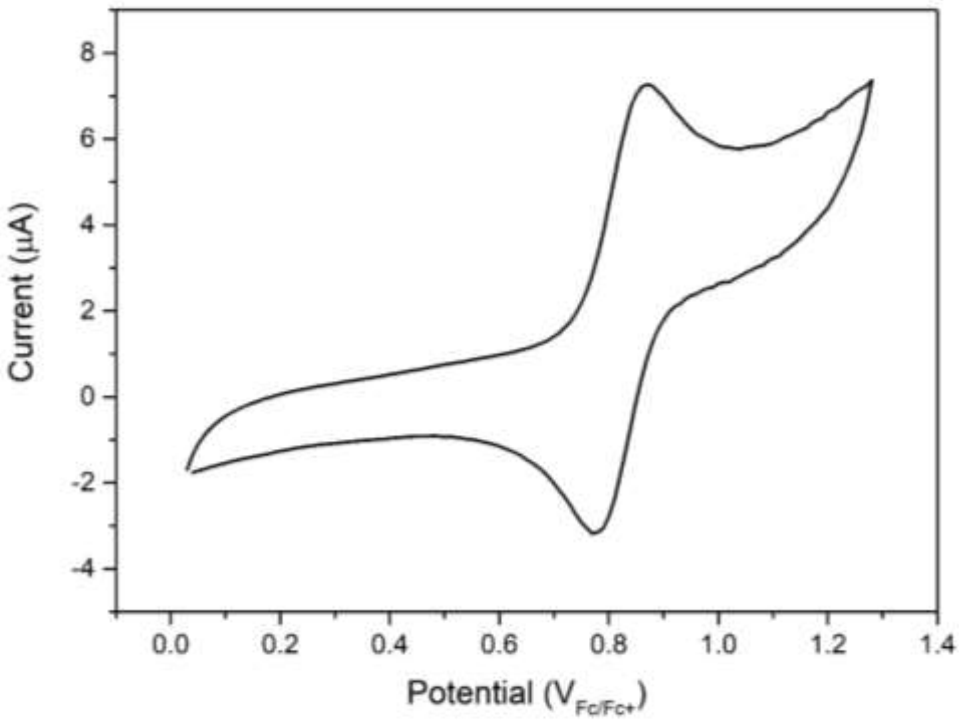

Figure S16. Cyclic voltammogram of complex **Fe-2** recorded with scan rate of 100 mV s^{-1}.

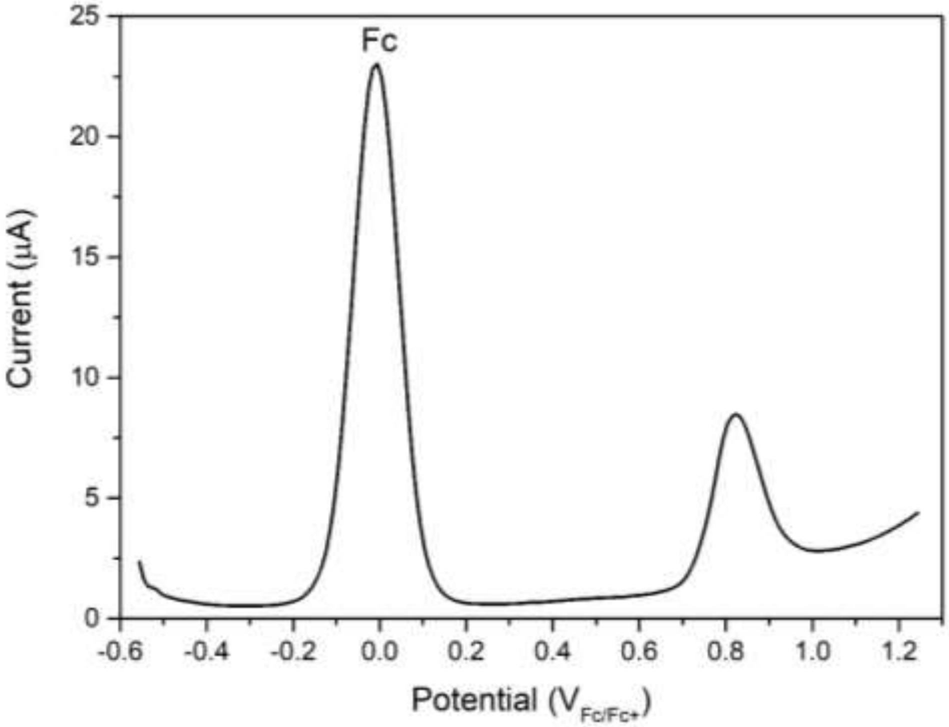

Figure S17. Differential pulse voltammogram of complex **Fe-2**.

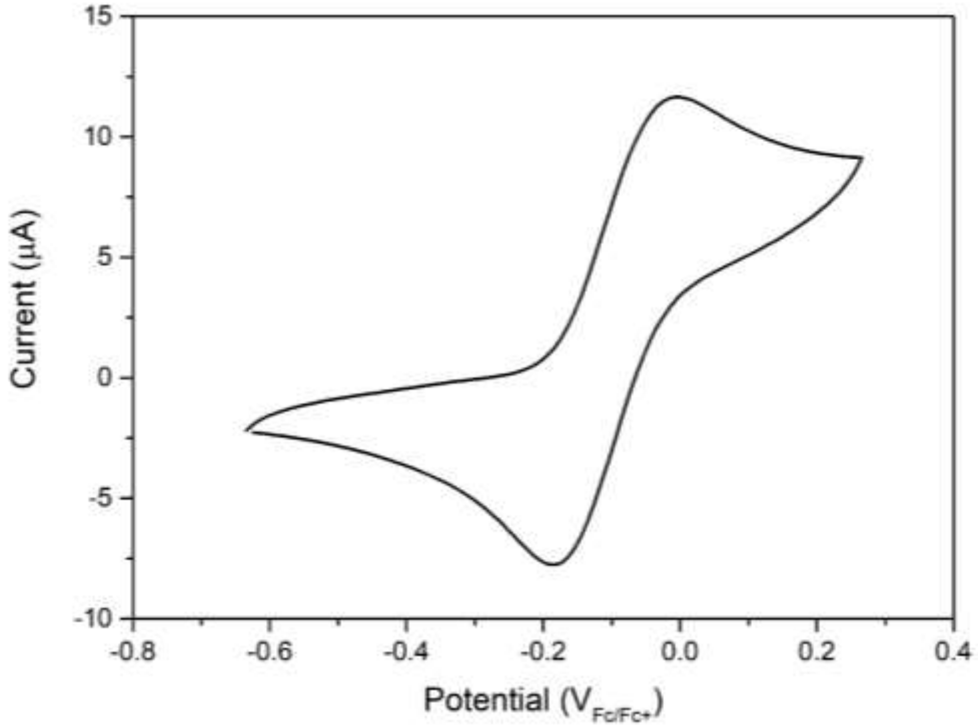

Figure S18. Cyclic voltammogram of complex **Co-2** recorded with scan rate of 100 mV s^{-1}.

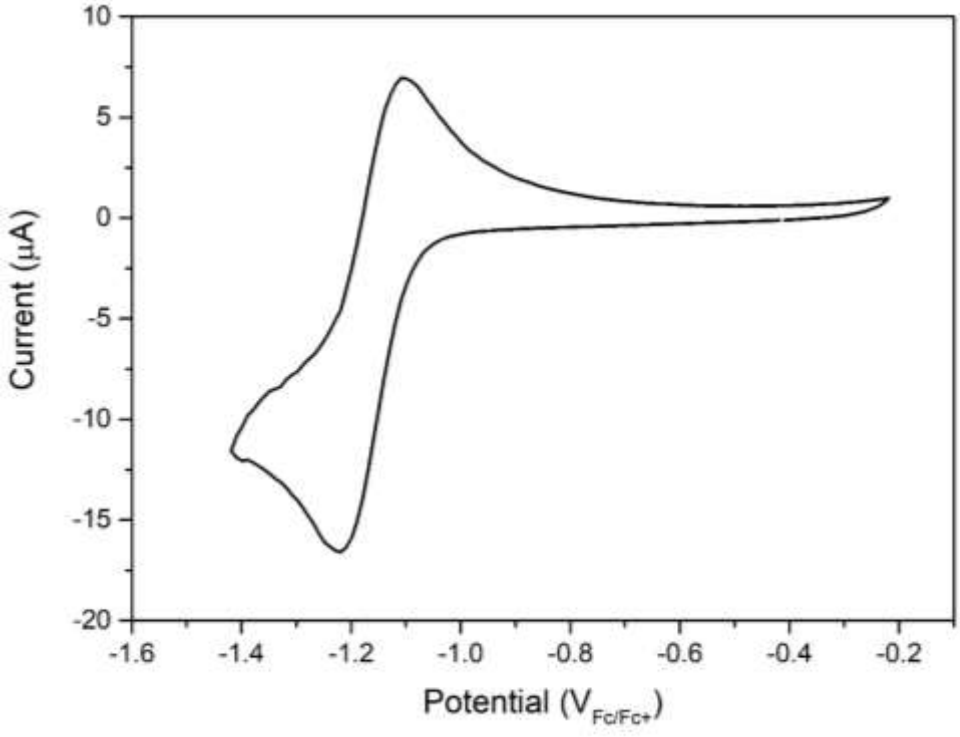

Figure S19. Cyclic voltammogram of complex **Co-2** recorded with scan rate of 100 mV s^{-1}.

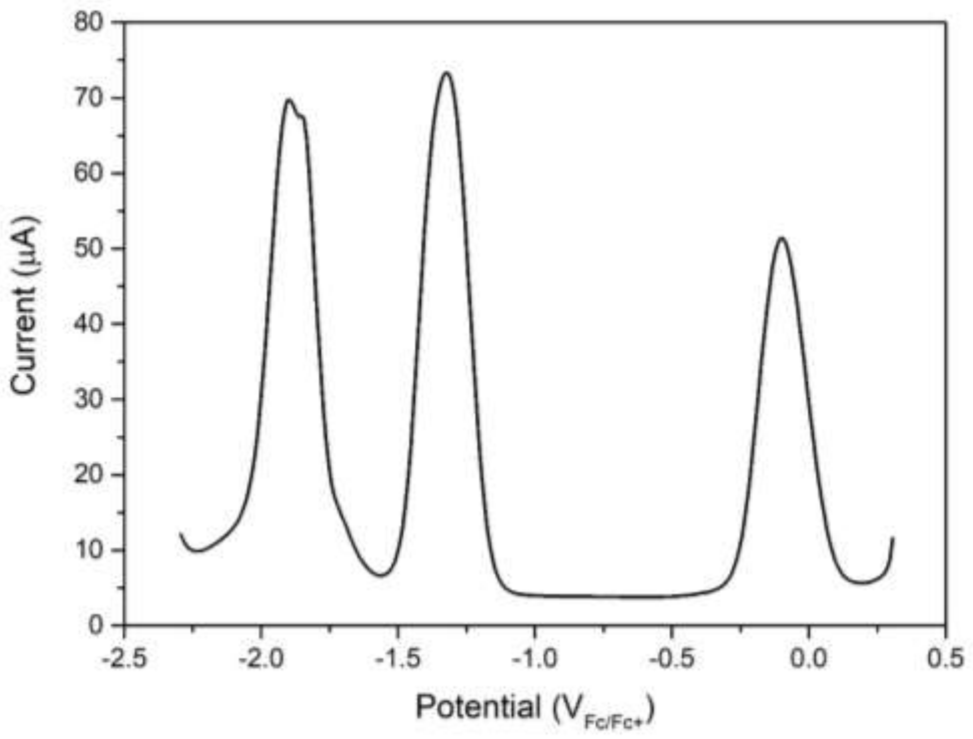

Figure S20. Differential pulse voltammogram of complex **Co-2**.

Electronic absorbance data were collected at room temperature in an acetonitrile solution for the metal complexes **Fe-1**, **Fe-2**, **Co-1**, and **Co-2**; while the spectrum for $\mathbf{L^1}$ was recorded in dichloromethane.

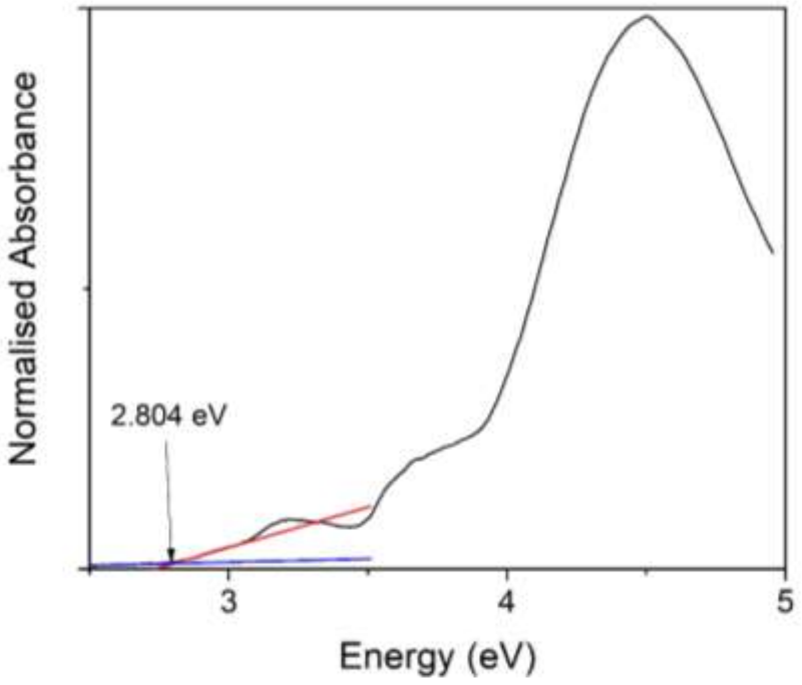

Figure S21. Electronic absorbance spectrum of $\mathbf{{}^1L}$.

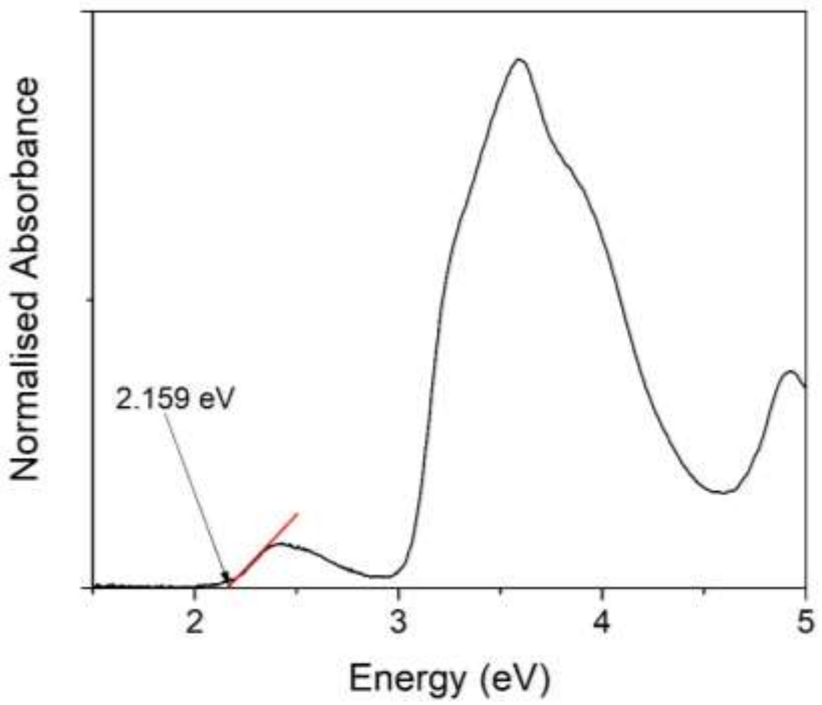

Figure S22. Electronic absorbance spectrum of **Fe-1**.

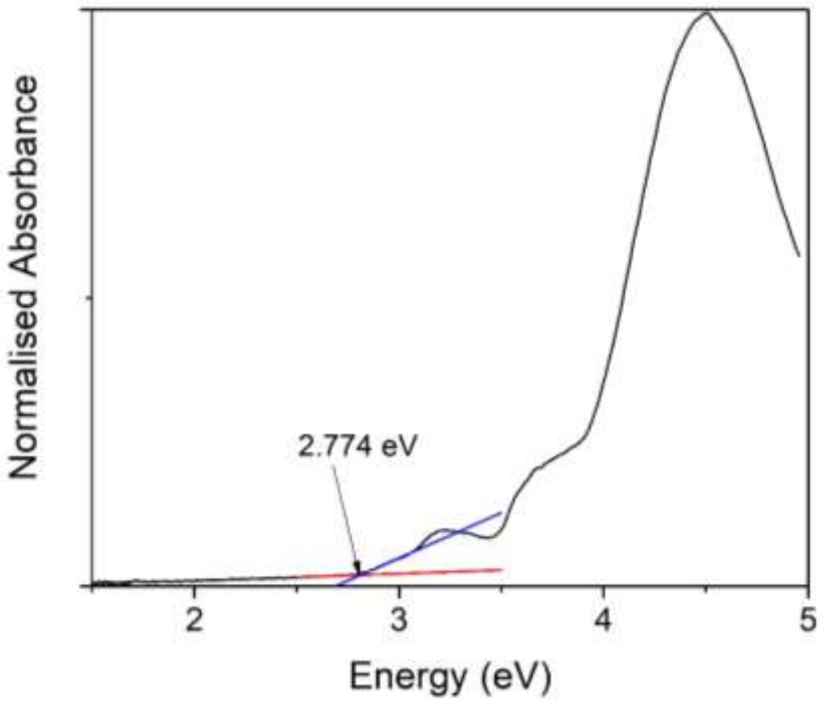

Figure S23. Electronic absorbance spectrum of **Co-1**.

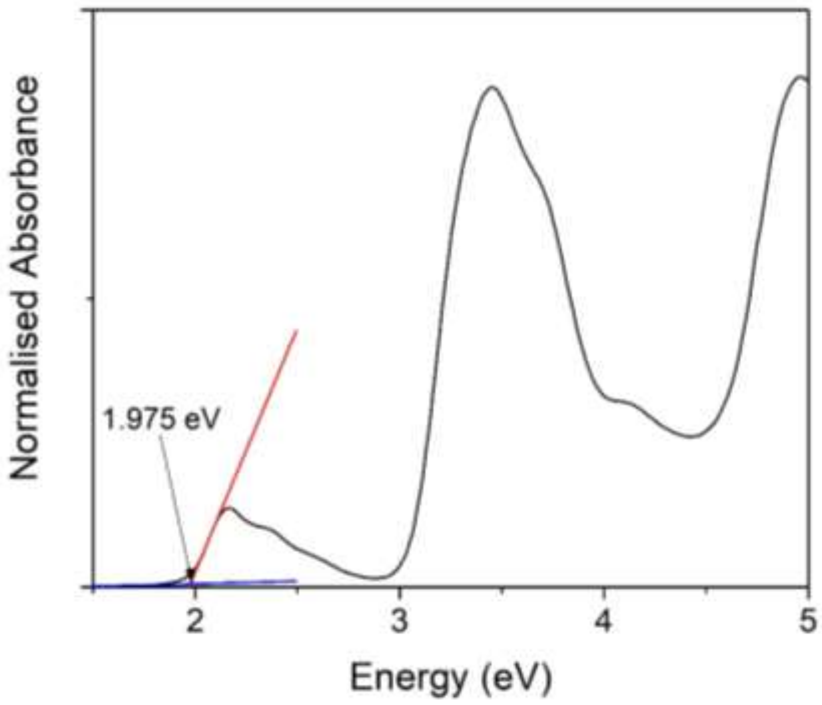

Figure S24. Electronic absorbance spectrum of **Fe-2**.

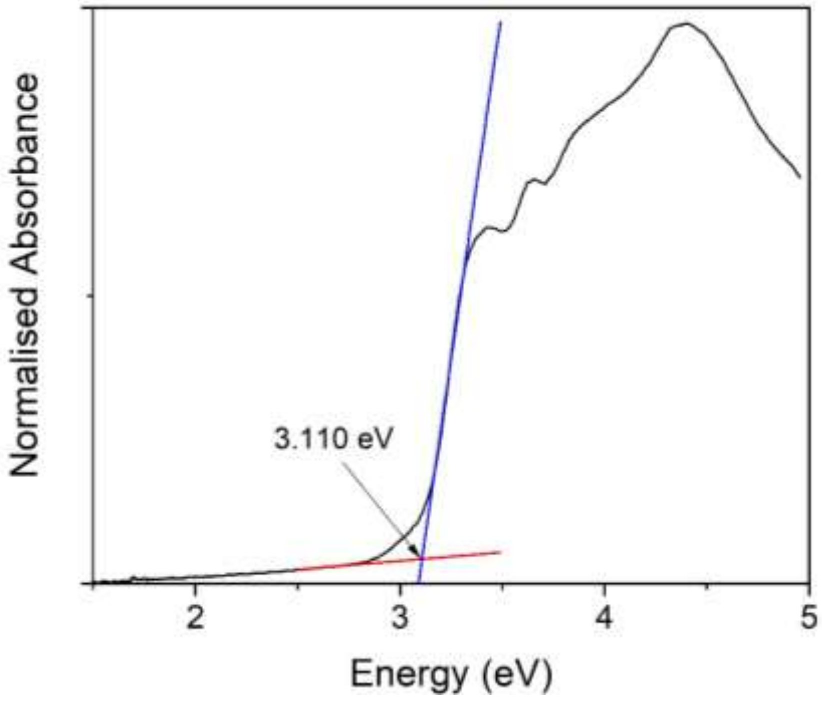

Figure S25. Electronic absorbance spectrum of **Co-2**.

b) Surface Characterisation

1. XPS. Experimental

XPS spectra were recorded on a Kratos AXIS ultra DLD spectrometer equipped with an Al Kα X-ray monochromatic source (1486.6 eV) and using 20 eV as pass energy. Binding energies were calibrated according to the C1s peak at 284.6 eV.

2. QCM. Experimental

Quartz Crystal Microbalance (QCM) measurements were carried out using a Stanford Research System instrument and employing AT-cut, α-quartz crystals with a resonant frequency of 5 MHz having circular gold electrodes patterned on both sides. The frequency change (Δf) for a QCM quartz resonator before and after the deposition process was determined. Taking into account the Sauerbrey equation:

$$\Delta f = -\frac{2f_0^2\Delta m}{A\rho_q^{1/2}\mu_q^{1/2}}$$

where f_0 is the fundamental resonant frequency of 5 MHz, Δm(g) is the mass change, A is the electrode area, ρ_q is the density of the quartz (2.65 g·cm^{-3}), and μ_q is the shear module (2.95·10^{11} dyn·cm^{-2}). From Δm and considering the surface of the resonator, the surface coverage, Γ, expressed as mol per square centimeter can be determined by considering the compound's molecular weight.

c) Conductance measurements

Propylene carbonate (PC), and 1,3,5-trimethylbenzene (TMB) were purchased from Sigma-Aldrich, UK.

Liquid sample cells made out of PTFE or PCTFE were used for all scanning tunnelling microscopy (STM) experiments. Prior to each experiment, the cells were thoroughly cleaned by sonicating them in three steps, first immersed in piranha solution, then in Milli-Q water, and finally in acetone, approximately 15 minutes each. Piranha solution was prepared by adding 30 w/w% hydrogen peroxide to concentrated sulfuric acid in a 1:3 ratio. !Caution—Piranha solution is a very strong oxidising agent and releases hazardous fumes. Never add concentrated sulfuric acid to hydrogen peroxide! Explosion risk when in contact with organic material!

All conductance experiments were carried out using STM gold tips, which were cut with scissors from a 99.99% pure gold wire 0.25 mm in diameter, purchased from Goodfellow Cambridge Ltd (England). For measurements carried out in polar solvents like PC, the tips were etched electrochemically and subsequently coated in Apiezon wax. The etching procedure involves suspending the gold wire into a 1:1 mixture of hydrochloric acid:ethanol solution. A second gold wire is placed around the STM tip in a ring, touching the surface of the solution to form a meniscus. Both wires are connected to a power supply, where the STM tip acts as the anode and the ring wire as the cathode. Upon applying a potential of approximately 5 V, gold at the anode dissolves into solution by reacting with chloride ions to form chloroaurate ions. The reaction continues for a few minutes until the bottom of the wire breaks off due to gravity and leaves behind a sharp, cone-shaped tip. The power supply is switched off, and the tip is rinsed with Milli-Q water and then coated with Apiezon wax. The wax is heated up on a fork-shaped soldering iron at 160 °C until it melts and the tip is pushed through the wax from the

bottom with the sharp end. The coated tip is then pulled away horizontally and allowed to cool to room temperature before being mounted in the STM scanner.

Gold substrates were purchased from Arrandee GmbH (Germany), which have a 250 nm gold (Au) layer on borosilicate glass with a 2.5 nm chromium adhesion layer in between. Before each experiment, the substrates were rinsed with acetone and then gently flame-annealed using a butane torch while placed on top of a silicon wafer to avoid bending of the glass. The substrate was heated until it started glowing bright orange, after which the flame is held for another ~5 seconds before removing it and allowing the substrate to cool for ~1 minute. This process is then repeated two more times, which yields large Au(111) terraces that are atomically flat.

Raw STMBJ data was converted into conductance-distance traces using either a Python script ('Cerberus') or a LabVIEW routine ('Leviathan'). Only data corresponding to the withdrawal portion of the experiment was used for analysis, and the approach data is discarded. Several thousand traces were collected for each molecule and presented without selection in logarithmically binned conductance histograms. Average conductance values were determined by fitting the histogram peaks with Gaussian curves.

- **Fe-1** dataset contains 5637 scans measured at 100 mV
- **Co-1** dataset contains 1135 scans measured at 700 mV
- **L^1** dataset contains 4378 scans measured at 300 mV
- **Fe-2** dataset contains 3697 scans measured at 100 mV
- **Co-2** dataset contains 2000 scans measured at 100 mV

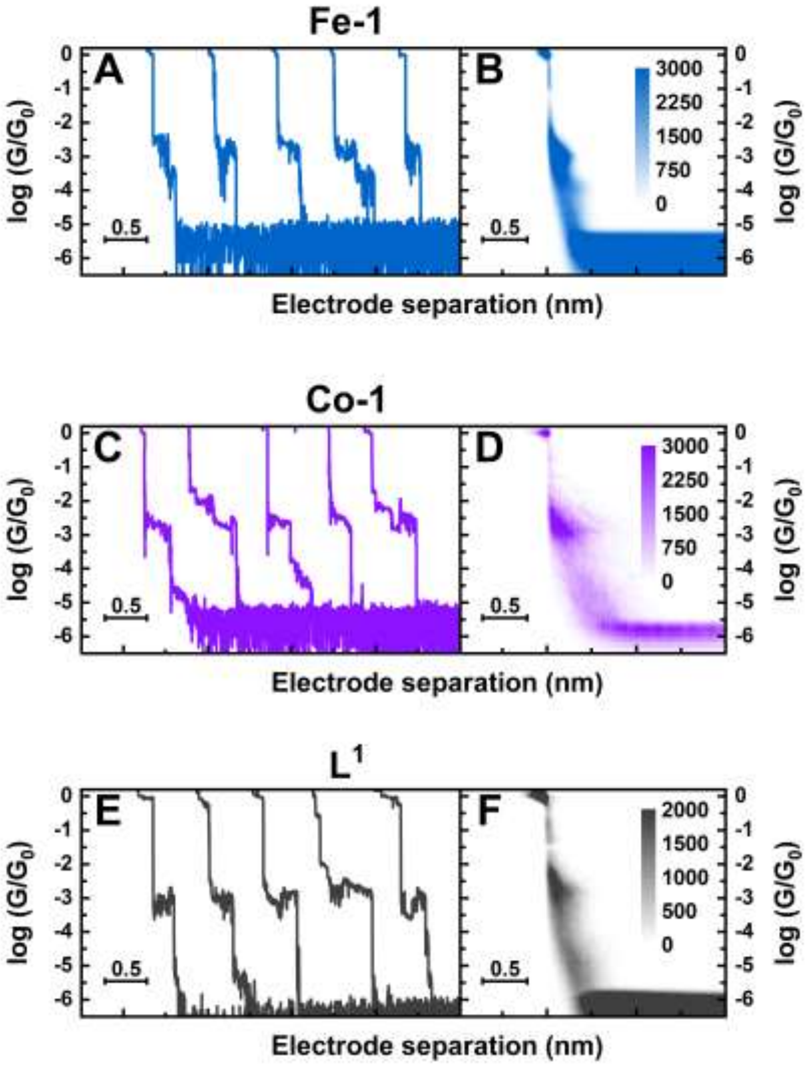

Figure S26. Representative conductance-distance traces along with two-dimensional histograms. (A,B) Dataset for **Fe-1**, (C,D) dataset for **Co-1**, and (E,F) dataset for **L^1**.

We also performed unsupervised clustering analysis on **Co-2** and **Fe-2** to better understand the distribution of conductance features in our datasets.[8] Clustering has been performed with a k-means algorithm, using a 28x28 feature space and tSNE dimensionality reduction. This feature space / clustering algorithm combination ensures high accuracy (FM > 0.85).[9]

In the case of **Co-2** the clustering algorithm enables the separation of the dataset in three clusters (see Figure S27). Cluster1 contains ~32% of the traces and shows only direct electrode tunnelling – no molecular junctions formed. The second and third cluster (respectively 33% and 35% of the traces) instead contain the data relative to molecular junctions. Cluster 2 is mostly made by traces with low conductance, while in Cluster 3 the traces contributing to the

high-conductance feature are isolated. In both cases, however, the electrode separation is not consistent with the full length of the molecular wire.

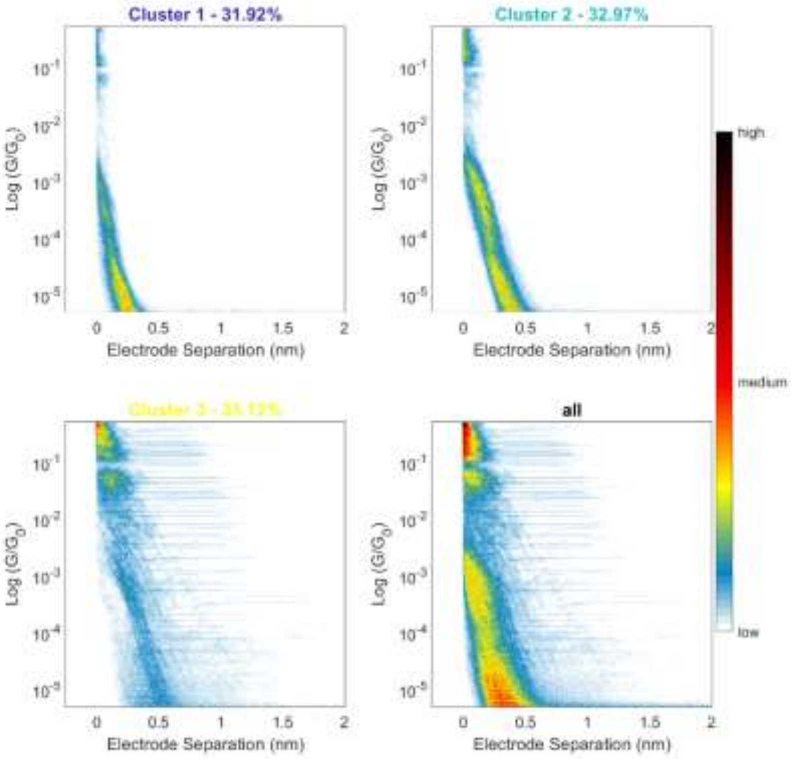

Figure S27. Unsupervised clustering analysis results for **Co-2**.

In the case of **Fe-2** the clustering algorithm returns two clusters with just direct tunnelling (a total of 73.5% of the whole dataset) and a third cluster where molecular junctions are isolated

(see Figure 28). In this case, there is good agreement between molecular length (end-to-end) and electrode separation, suggesting the formation of extended molecular junctions.

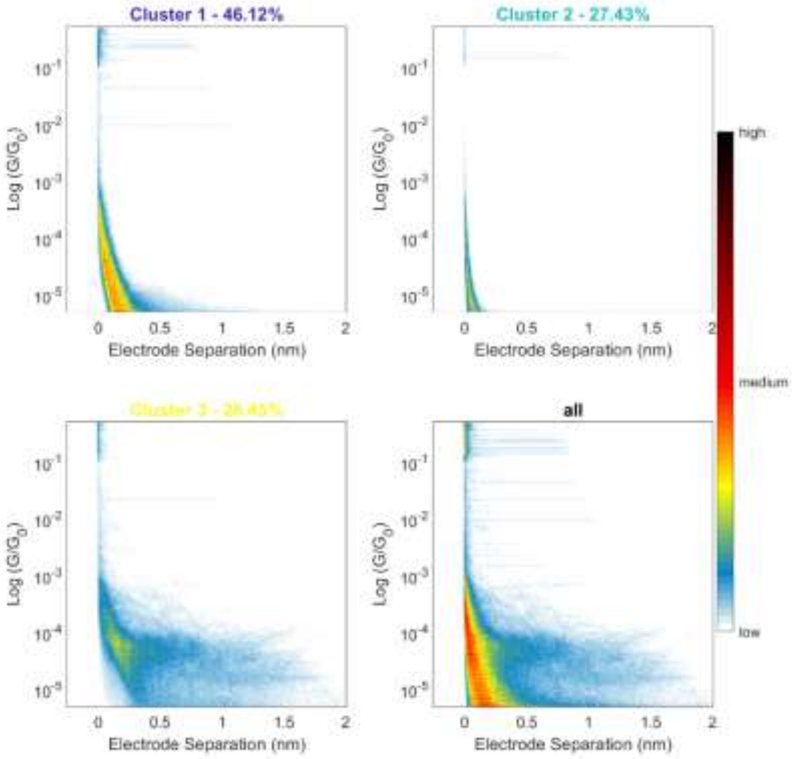

Figure S28. Unsupervised clustering analysis results for **Fe-2**.

Overall, the clustering analysis does not isolate any feature that suggest transport through the entire length of the molecular wire in the **Co-2** dataset, but it returns strong evidence for robust junction formation in **Fe-2** (albeit in a low overall percentage of 26.5%).

d) Theoretical details

Figure S29 bellow shows the relaxed structure of molecules **Fe-1** (a), **Fe-2** (b), **Co-1** (c), and **Co-2** (d) with counterions shown.

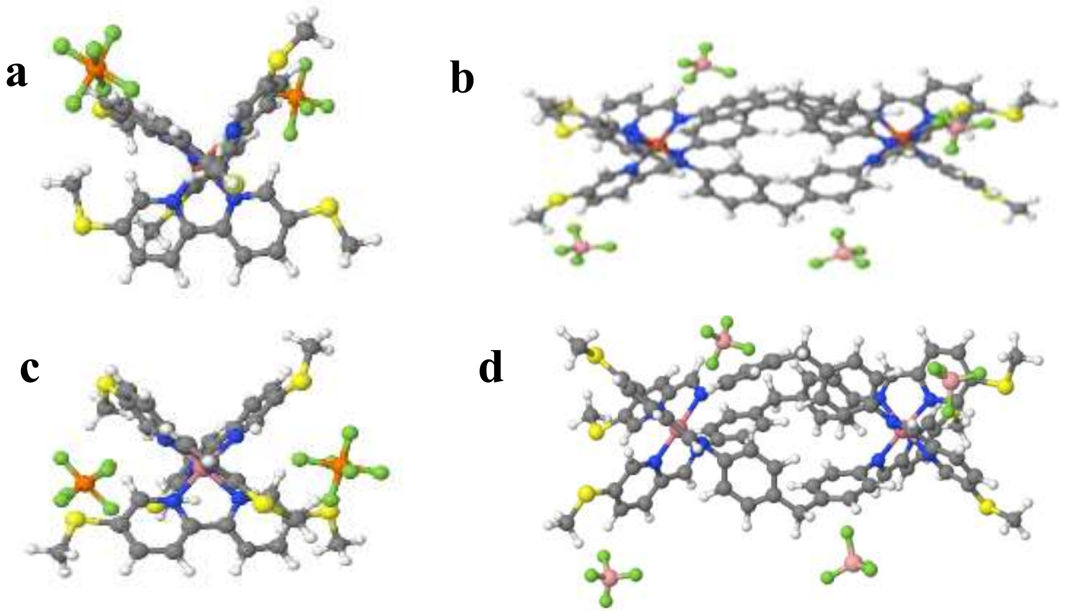

Figure S29. DFT-based relaxed gas-phase, (a) and (b) configurations represent the central Iron atom (**Fe-1**and **Fe-2**) at the core respectively. (c) and (d) configurations represent the central cobalt atom (**Co-1** and **Co-2**) at the core respectively.

1. Modelling charge transport through gold/metal-molecule/gold junctions

We computed transmission curves for **Fe-1** and compared it to **Co-1** and the double metal core compounds **Fe-2** and **Co-2**, with all of these respective molecules attached to gold electrodes via SMe anchor groups and geometries optimised. Then we used density functional theory (DFT) combined with the quantum transport code Gollum to compute the transmission coefficients (see Method section for details). We then evaluate the conductance value from the Landauer formula at room temperature $G = G_0 T(E_F)$, where E_F is the Fermi energy of the electrodes, within the HOMO-LUMO (Highest Occupied Molecular Orbital- Lowest Unoccupied Molecular Orbital) gap over a range of energies. Since these compounds have six-thiomethyl groups (Figure S29) there are different contact possibilities to the electrodes. We examined geometries of the molecules attached to the electrodes at all the different possible connection points (Figure S31). The first connectivity (blue arrow) connects the electrodes across the same ligand, the others (red and yellow arrow) are connected through two different ligands, with electronic charge then transmitting passing from one ligand to adjacent ones. By comparing the transmission coefficients of these connections for **Fe-1** and **Co-1** (Figure S32-S34), we found that the blue connectivity (same ligand) gives better agreement with the experimental measurements. Similar behaviour is found for the double metal core **Fe-2** and **Co-2** targets (see figure S25-S27), where the blue connectivity shows good agreement with the experimental values.

Figure 4 of the manuscript shows the electrical transmission of the junctions through the same ligand for Fe**-1, Co-1**, **Fe-2** and **Co-2** molecules, and the DFT predicted Fermi energy E_F^0 lies close to the LUMO resonance. The values of the electrical conductance of the single-metal core are higher than the double-metal core as expected due to the much greater length of the latter. For the junctions with a cobalt core (**Co-1** and **Co-2**) the electronic structures are spin polarised,

so we compute the total $T_e= T_e\uparrow+T_e\downarrow/2$ from the transmission coefficient of majority (↑) and minority (↓) spins.

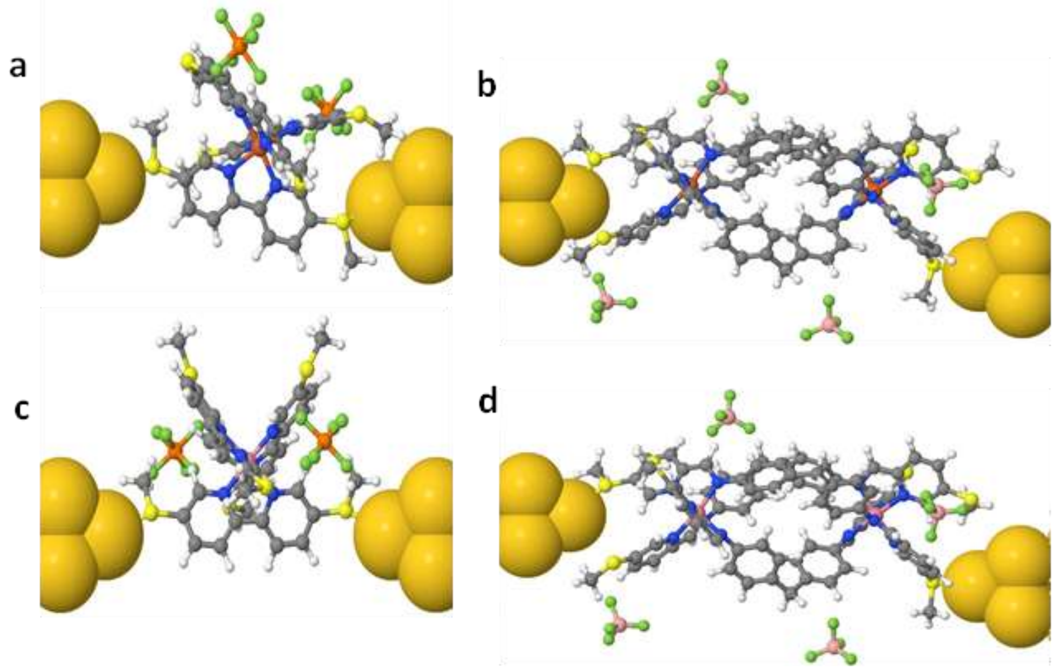

Figure S30. DFT-based optimised configurations of the metal helix wires (a) **Fe-1**, (b) **Fe-2**, (c) **Co-1** and (d) **Co-2.** These molecules in these junctions are connected to the two gold electrodes and correspond to the transmission curves shown in figure 4 in the manuscript.

Figure S30 shows relaxed junctions for transmission curves shown in Figure 4 of the manuscript. However, to further investigate the effect of connectivities on electrical conductance of the molecules we calculated the electrical conductance of other possible configurations. These connectivities are schematically illustrated in the following simple model (Figure S31) with different metal containing molecular bridges (**Fe-1**, **Fe-2**, **Co-1** and **Co-2**) attached to the different thiomethyl units.

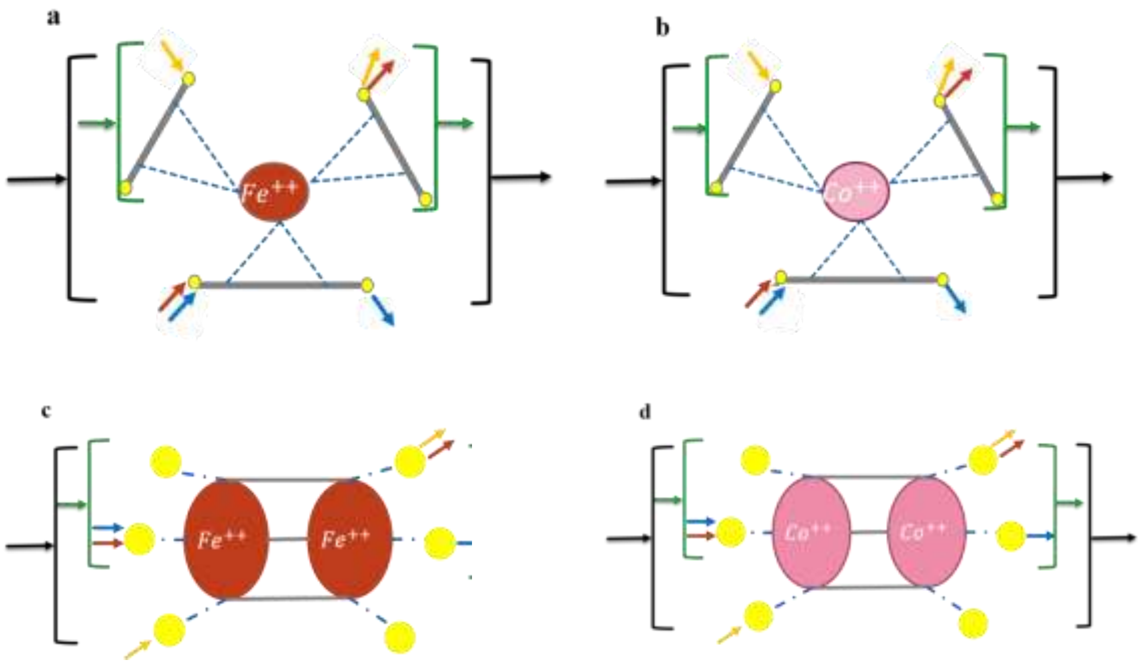

Figure S31. The simple model of the single metal core molecules (**Fe-1** and **Co-1**) and the double metal core molecules (**Fe-2** and **Co-2**) with different connectivities to the gold electrode. (a) the structure of a single iron atom at the core (**Fe-1**), (b) the structure of a single cobalt atom at the core (**Co-1**), these cores are connected to the three different helix-wire (solid grey lines) with six-thiomethyl units via nitrogen atoms (blue dashed line). (c) The structure of double iron atoms at the core (**Fe-2**), (d) the structure of double cobalt atoms at the core (**Co-2**). Three are three possible connectivities are shown with different arrows colours (blue, red and yellow). The blue arrow shows the connection of the electrodes through the same ligand (named as **I**). The red and yellow arrows show the connection of the electrodes from one ligand to another one (named **II** and **III**) with larger tip-tip distances. The green arrow shows 2 thiomethyl anchors connected to the electrode and the black arrow indicates that 3 thiomethyl are connected. The electrical conductance of **Fe-1**, **Co-1, Fe-2** and **Co-2** with different connectivities is shown in figure S32-37.

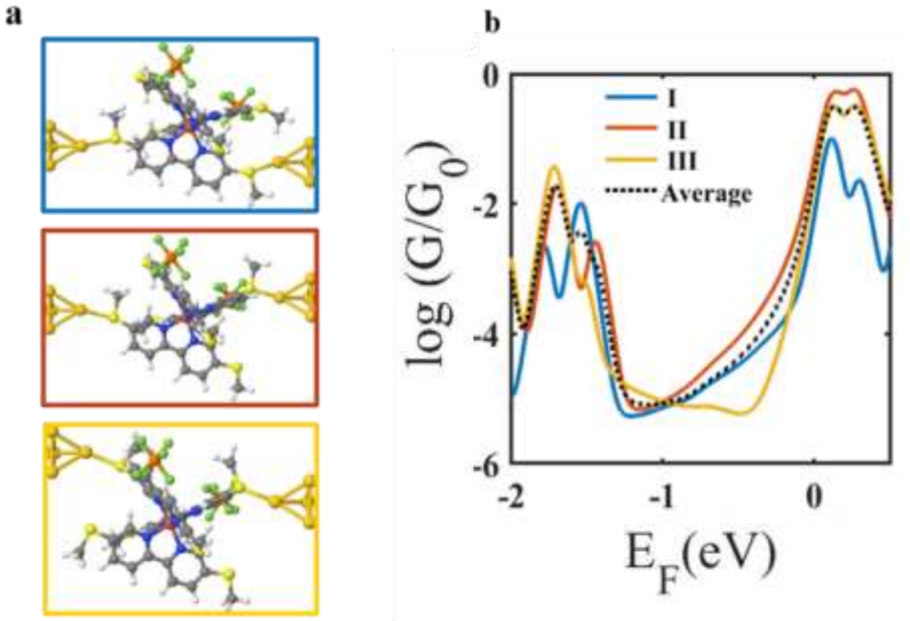

Figure S32. DFT based electrical conductance of **Fe-1** for three different connectivities to the gold electrode as shown in figure S30-a, (a) relaxed structures of **Fe-1** between two electrodes connected through three different thiomethyl units as illustrated, (b) DFT-based electrical conductance against Fermi energy of the electrode. The black dashed line is the average of these different connectivities.

Figure S32 and 34 shows the electrical conductance of **Fe-1**, **Co-1** where the electrodes are attached to multiple thiomethyl units as shown in figure S31 (Green arrow shows 2-SMe connection and black arrow shows 3-SMe connection). Based on DFT calculations when 2-SMe (two thiomethyl groups are in contact with the surface) is relaxed the resulting geometry is the 3-SMe configuration (three thiomethyl groups making contact with the surface). Overall, the single-core configuration shows that the 3-SMe connection has a higher electrical conductance than single thiomethyl connection. While for the complex double metal core configuration, the electrical conductance around the zero Fermi energy remains unchanged.

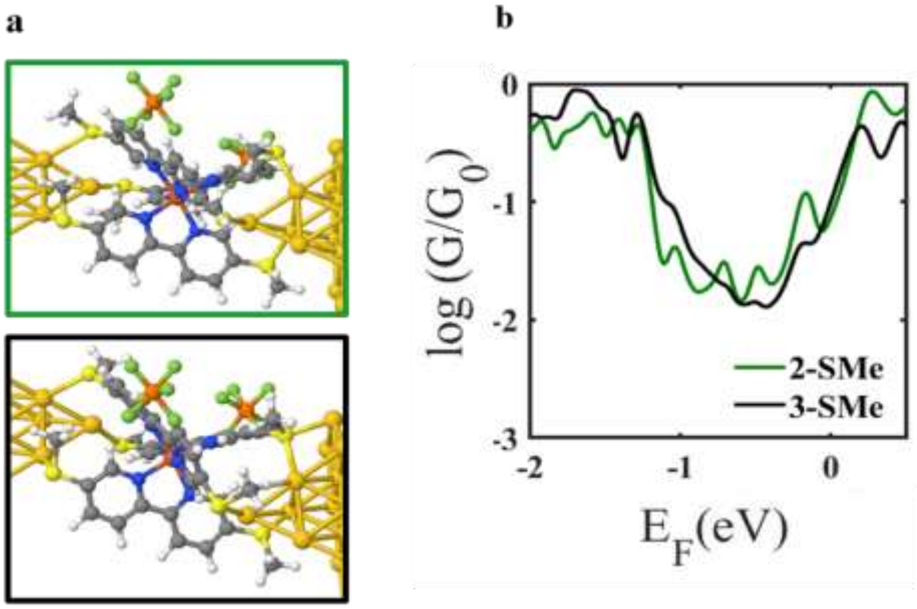

Figure S33. DFT-based electrical conductance of **Fe-1** with multiple thiomethyl units connecting to each gold electrode as shown schematically in figure S20-a, (a) relaxed structures of the **Fe-1** molecules between two electrodes connected with multiple thiomethyl units, (b) DFT-based electrical conductance against Fermi energy of the electrode.

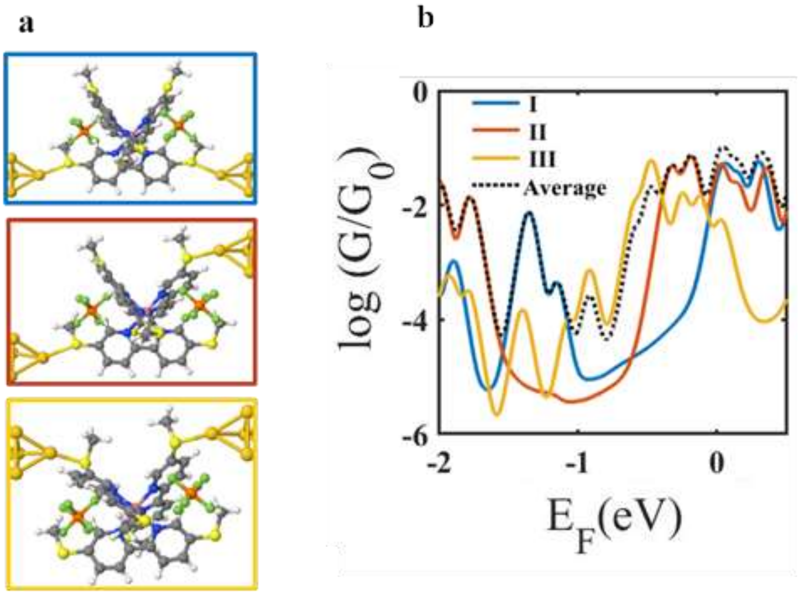

Figure S34. DFT based electrical conductance of **Co-1** for three different connectivities to the gold electrode as shown in figure S30-b, (a) relaxed structures of the **Co-1** molecule between two electrodes connected through three different thiomethyl units. (b) DFT-based average spin polarised electrical conductance against Fermi energy of the electrode and the black dashed line is the average of these different connectivities.

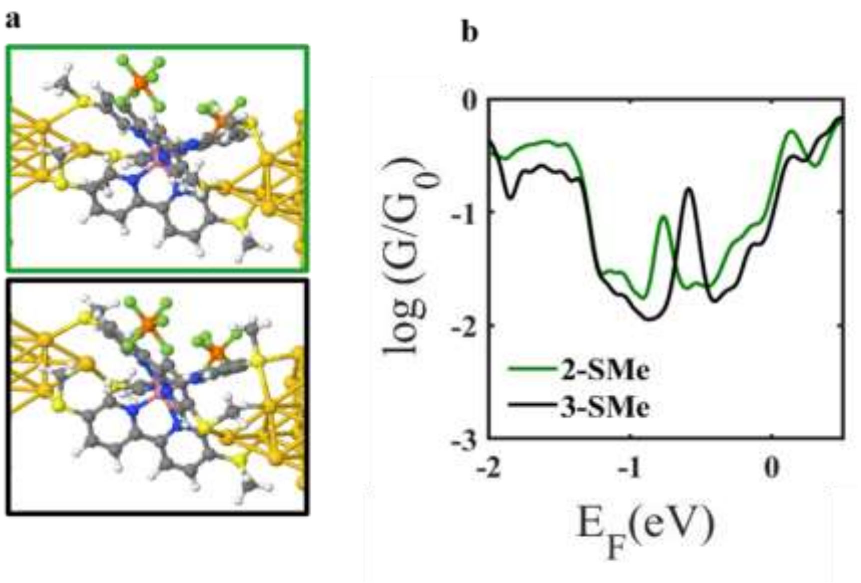

Figure S35. DFT-based electrical conductance of **Co-1** with multiple thiomethyl unit connections to each gold electrode as shown in figure S30-b, (a) relaxed structures of the **Co-1** molecules between two electrodes connected with multiple thiomethyl units, (b) DFT-based average spin polarised electrical conductance against Fermi energy of the electrode.

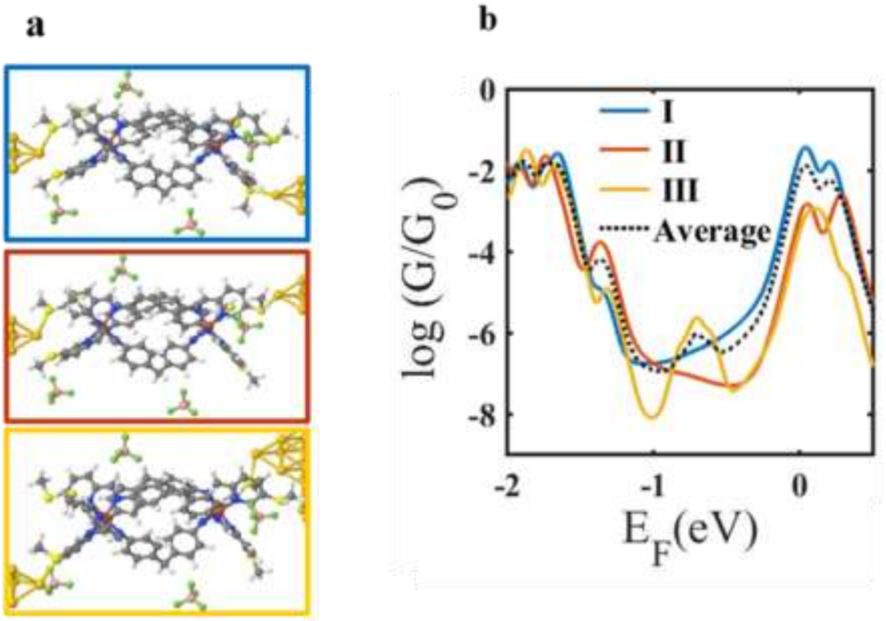

Figure S36. DFT based electrical conductance of **Fe-2** for three different connectivities to the gold electrode as in figure S30-c, (a) relaxed structures of the **Fe-2** molecule between two electrodes connected to three different thiomethyl units. (b) DFT-based electrical conductance against Fermi energy of the electrode and the black dashed line is the average of these different connectivities.

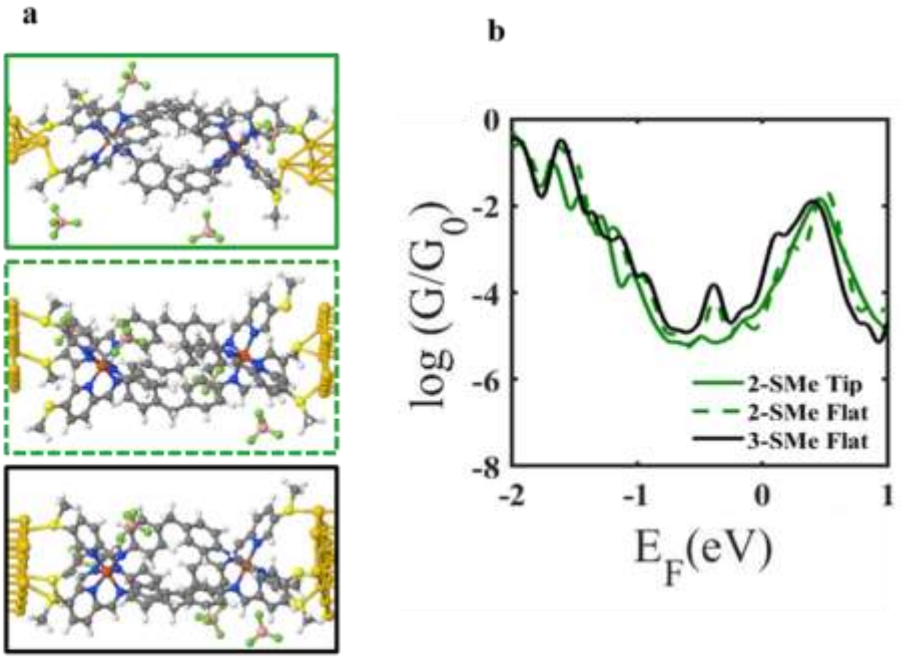

Figure S37. DFT-based electrical conductance of **Fe-2** for multiple thiomethyl units connecting to different gold electrode shapes as shown, (a) relaxed structures of the **Fe-2** molecules between two electrodes connected with multiple thiomethyl units, (b) DFT-based electrical conductance against Fermi energy of the electrode.

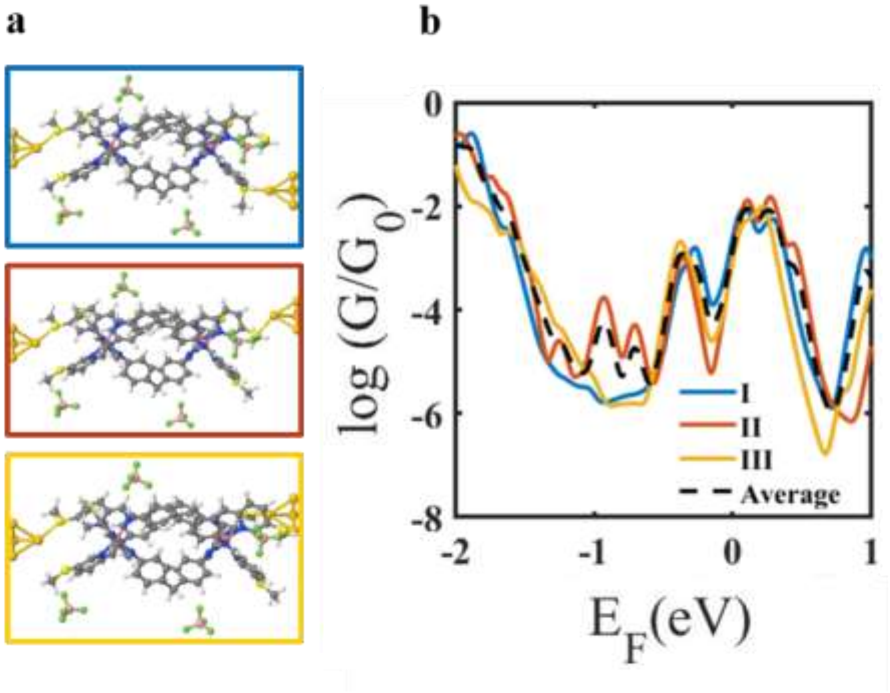

Figure S38. DFT-based electrical conductance of **Co-2** for three different connectivities to the gold electrode as in figure S30-d, (a) relaxed structures of the **Co-2** molecule between two electrodes connected to three different thiomethyl units. (b) DFT-based average spin polarised electrical conductance against Fermi energy of the electrode and the black dashed line is the average of these different connectivities.

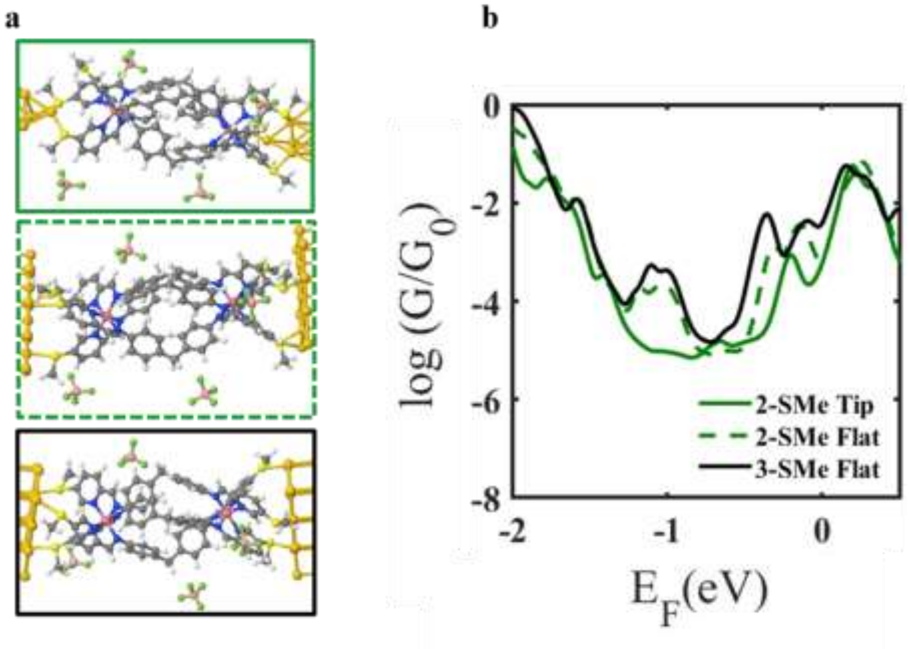

Figure S39. DFT-based electrical conductance of **Co-2** for multiple SMe units connecting to different gold electrode shapes, (a) relaxed structures of the **Co-2** molecules between two electrodes connected with multiple thiomethyl units, (b) DFT-based average spin-polarised electrical conductance against Fermi energy of the electrode.

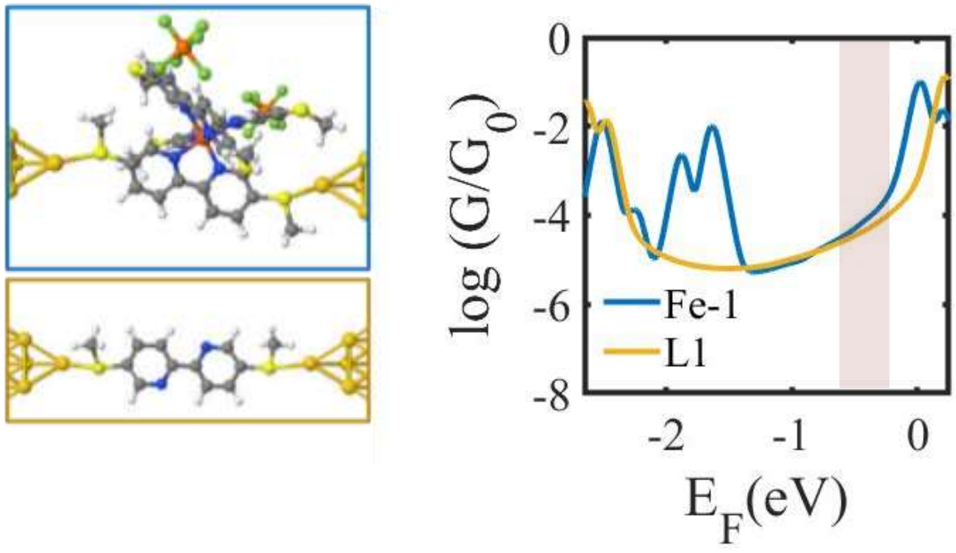

Figure S40. Relaxed structures of the complex **Fe-1** and **L1** molecules between two electrodes (left), DFT-based electrical conductance against Fermi energy of the electrode (right).

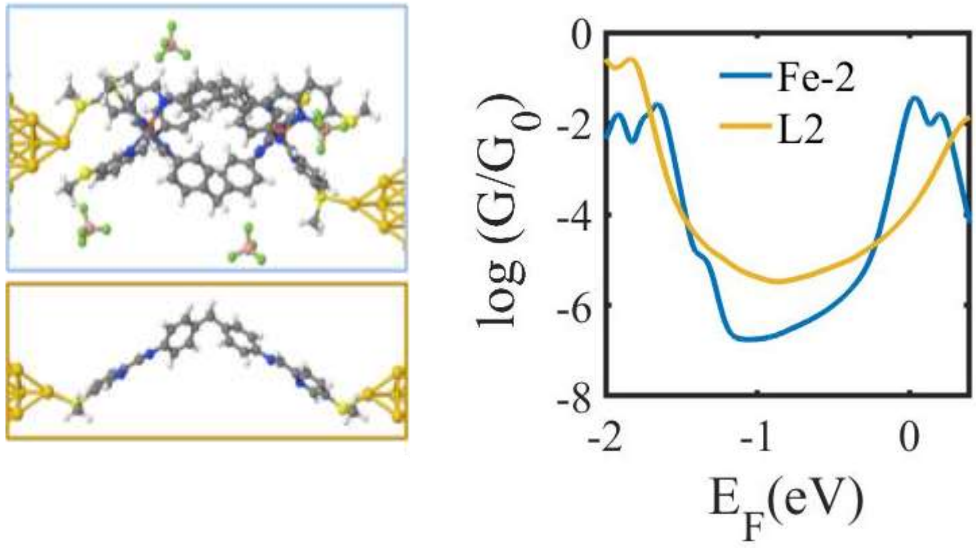

Figure S41. Relaxed structures of the complex **Fe-2** and **L2** molecules between two electrodes (left), DFT-based electrical conductance against Fermi energy of the electrode (right).

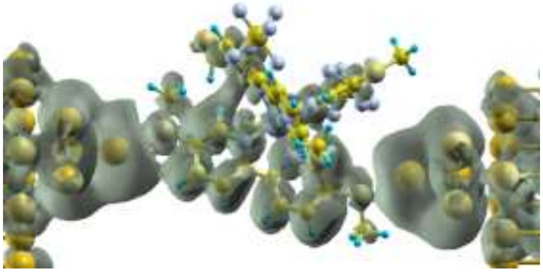

Figure S42. Local density of state (LDOS) calculation for structure I of **Fe-1** around LUMO resonance.

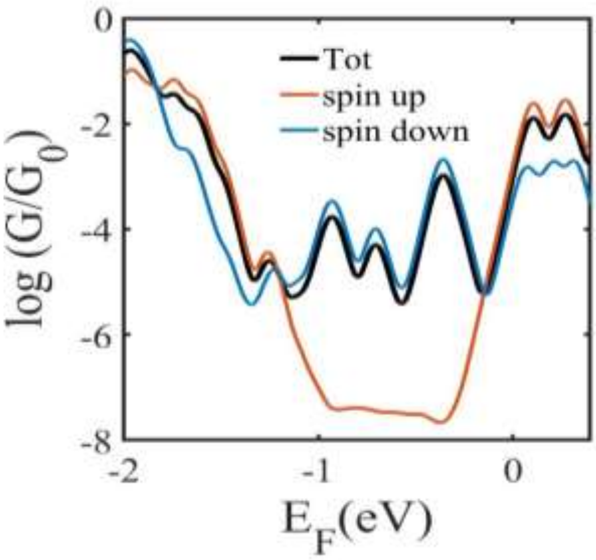

Figure S43. Spin up, down, and total transmission (spin up + spin down)/2 for the structure II of Figure S38-a.

2. Computational methods

DFT calculations: The optimized geometry and ground state Hamiltonian and overlap matrix elements of each structure was self-consistently obtained using the SIESTA[10] implementation of density functional theory (DFT). SIESTA employs norm-conserving pseudo-potentials to

account for the core electrons and linear combinations of atomic orbitals to construct the valence states. The generalized gradient approximation (GGA) of the exchange and correlation functional is used with the Perdew-Burke-Ernzerhof parameterization (PBE) a double-ζ polarized (DZP) basis set, a real-space grid defined with an equivalent energy cut-off of 250 Ry. The geometry optimization for each structure is performed until the forces are smaller than 10 meV/Å.

Transport calculations:

The mean-field Hamiltonian obtained from the converged DFT calculation or a tight-binding Hamiltonian (using single orbital energy site per atom with Hückel parameterisation) was combined with our home-made implementation of the non-equilibrium Green's function method, GOLLUM[11], to calculate the phase-coherent, elastic scattering properties of each system consisting of left gold (source) and right gold (drain) leads and the scattering region (**Fe-1**, **Co-1, Fe-2** and **Co-2** molecules). The transmission coefficient[12] $T(E)$ for electrons of energy E (passing from the source to the drain) is calculated via the relation:

$$T(E) = Trace\ (\Gamma_R(E) G^R(E) \Gamma_L(E) G^{R\dagger}(E)$$

In this expression, $\Gamma_{L,R}(E) = i\left(\Sigma_{L,R}(E) - \Sigma_{L,R}{}^{\dagger}(E)\right)$ describe the level broadening due to the coupling between left (L) and right ® electrodes and the central scattering region, $\Sigma_{L,R}(E)$ are the retarded self-energies associated with this coupling and $G^R(E) = (ES - H - \Sigma_L - \Sigma_R)^{-1}$ is the retarded Green's function, where H is the Hamiltonian and S is overlap matrix. Using obtained transmission coefficient $T(E)$, the conductance could be calculated using the Landauer formula $(G = G_0 \int dE\ T(E)(-\partial f/\partial E))$ where $G_0 = 2e^2/h$ is the conductance quantum. $f(E) = (1 + exp((E - EF)/k_B T))^{-1}$ is the Fermi−Dirac distribution function, T is the temperature, and $k_B = 8.6 \times 10^{-5}\ eV/K$ is Boltzmann's constant.

d) References

1. He, Z.; Li, M.; Que, W.; Stang, P. J. Self-assembly of metal-ion-responsive supramolecular coordination complexes and their photophysical properties. *Dalton Transactions* **2017,** 46 (10), 3120-3124 DOI: 10.1039/C7DT00174F.

2. Li, J.; Lynch, M. P.; DeMello, K. L.; Sakya, S. M.; Cheng, H.; Rafka, R. J.; Bronk, B. S.; Jaynes, B. H.; Kilroy, C.; Mann, D. W.; Haven, M. L.; Kolosko, N. L.; Petras, C.; Seibel, S. B.; Lund, L. A. In vitro and in vivo profile of 2-(3-di-fluoromethyl-5-phenylpyrazol-1-yl)-5-methanesulfonylpyridine, a potent, selective, and orally active canine COX-2 inhibitor. *Bioorganic & Medicinal Chemistry* **2005,** 13 (5), 1805-1809 DOI: https://doi.org/10.1016/j.bmc.2004.11.048.

3. Dolomanov, O. V.; Bourhis, L. J.; Gildea, R. J.; Howard, J. A. K.; Puschmann, H. OLEX2: a complete structure solution, refinement and analysis program. *Journal of Applied Crystallography* **2009,** 42, 339-341 DOI: 10.1107/s0021889808042726.

4. Sheldrick, G. M. *SHELXL: Suite of Programs for Crystal Structure Analysis*, Tammanstrasse 4: Gottingen, 1998.

5. Vincent, K. B.; Gluyas, J. B. G.; Gückel, S.; Zeng, Q.; Hartl, F.; Kaupp, M.; Low, P. J. Tetrakis(ferrocenylethynyl)ethene: Synthesis, (Spectro)electrochemical and quantum chemical characterisation. *Journal of Organometallic Chemistry* **2016,** 821, 40-47 DOI: https://doi.org/10.1016/j.jorganchem.2016.04.018.

6. Barrière, F.; Camire, N.; Geiger, W. E.; Mueller-Westerhoff, U. T.; Sanders, R. Use of Medium Effects to Tune the ΔE1/2 Values of Bimetallic and Oligometallic Compounds. *Journal of the American Chemical Society* **2002,** 124 (25), 7262-7263 DOI: 10.1021/ja020309d.

7. Richert, S. A.; Tsang, P. K. S.; Sawyer, D. T. Ligand-centered redox processes for manganese, iron and cobalt, MnL_3, FeL_3, and CoL_3, complexes (L = acetylacetonate, 8-quinolinate, picolinate, 2,2'-bipyridyl, 1,10-phenanthroline) and for their tetrakis(2,6-dichlorophenyl)porphinato complexes[M(Por)]. *Inorganic Chemistry* **1989,** 28 (12), 2471-2475 DOI: 10.1021/ic00311a044.

8. Cabosart, D.; Abbassi, M. E.; Stefani, D.; Frisenda, R.; Calame, M.; Zant, H. S. J. v. d.; Perrin, M. L. A reference-free clustering method for the analysis of molecular break-junction measurements. *Applied Physics Letters* **2019,** 114 (14), 143102 DOI: 10.1063/1.5089198.

9. El Abbassi, M.; Overbeck, J.; Braun, O.; Calame, M.; van der Zant, H. S. J.; Perrin, M. L. Benchmark and application of unsupervised classification approaches for univariate data. *Communications Physics* **2021,** 4 (1), 50 DOI: 10.1038/s42005-021-00549-9.

10. Soler, J. M.; Artacho, E.; Gale, J. D.; Garcia, A.; Junquera, J.; Ordejon, P.; Sanchez-Portal, D. The SIESTA method for ab initio order-N materials simulation. *Journal of Physics: Condensed Matter* **2002,** 14 (11), 2745-2779 DOI: 10.1088/0953-8984/14/11/302.

11. Ferrer, J.; Lambert, C. J.; García-Suárez, V. M.; Manrique, D. Z.; Visontai, D.; Oroszlany, L.; Rodríguez-Ferradás, R.; Grace, I.; Bailey, S. W. D.; Gillemot, K.; Hatef, S.; Algharagholy, L. A. GOLLUM: a next-generation simulation tool for electron, thermal and spin transport. *New Journal of Physics* **2014,** 16 (9), 093029.

12. Sadeghi, H. Theory of electron, phonon and spin transport in nanoscale quantum devices. *Nanotechnology* **2018,** 29 (37), 373001 DOI: 10.1088/1361-6528/aace21.
